# Silver Telluride Colloidal Quantum Dot Solid for Fast Extended Shortwave Infrared Photodetector

**DOI:** 10.1002/advs.202407453

**Published:** 2024-10-07

**Authors:** Yongnam Ahn, So Young Eom, Gahyeon Kim, Jin Hyeok Lee, Beomkwan Kim, Dongeon Kim, Min‐Jae Si, Minjung Yang, Yujin Jung, Bo Seon Kim, Yoon Jang Chung, Kwang Seob Jeong, Se‐Woong Baek

**Affiliations:** ^1^ Department of Chemical and Biological Engineering Korea University Seoul 02841 Republic of Korea; ^2^ Department of Chemistry Korea University Seoul 02841 Republic of Korea; ^3^ School of Chemical Engineering Yeungnam University 280 Daehak‐ro Gyeongsan Gyeongbuk 38541 Republic of Korea

**Keywords:** colloidal quantum dot (CQD), extended SWIR, fast photoresponse, photodetector, silver chalcogenide

## Abstract

Extended shortwave infrared (eSWIR) photodetectors that employ solution‐processable semiconductors have attracted attention for use in applications such as ranging, night vision, and gas detection. Colloidal quantum dots (CQDs) are promising materials with facile bandgap tunability across the visible‐to‐mid‐infrared wavelengths. However, toxic elements, such as Hg and Pb, and the slow response time of CQD‐based IR photodetectors, limit their commercial viability. This article presents a novel eSWIR photodetector that is fabricated using silver telluride (Ag_2_Te) CQDs. Effective thiolate ligand exchange enables a lower trap density and improved carrier mobility in CQD solids. Furthermore, a vertical p‐n photodiode architecture with a favorable energy‐level landscape is utilized to facilitate charge extraction, resulting in a fast, room‐temperature‐operable, and toxic‐element‐free CQD photodetector. The best eSWIR Ag_2_Te CQD photodetector exhibits a fall time of 72 ns, representing the fastest response time among all prior CQD‐based eSWIR photodetectors, including those containing toxic elements, such as Pb and Hg.

## Graphical abstract

Thiolate ligands effectively passivate the surfaces of Ag_2_Te colloidal quantum dots (CQDs), improving the electrical properties of the CQD solids. Furthermore, precise energy‐level modification enables the creation of an efficient heterojunction combined with a vertical p‐n photodiode architecture, achieving a nanosecond‐level photoresponse.

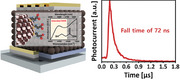

## 1 Introduction

Extended shortwave infrared (eSWIR) photodetectors offer considerable market potential for a wide range of applications, including surveillance, night vision, and chemical analysis.^[^ ^1^ ^]^ Particularly, molecules of gases such as CO_2_ (2.0 µm), CH_4_ (1.7 µm), and CO (1.6 µm) can be detected via low‐bandgap semiconductor‐based photodetection.^[^ ^2^ ^]^ Current commercial eSWIR photodetectors rely on bulk semiconductors, such as InGaAs, HgCdTe, InAs, InSb, and type II superlattices.^[^ ^3^ ^]^ However, high cost and low‐temperature operation present challenges to their widespread adoption and miniaturization.^[^ ^4^ ^]^

Alternatively, eSWIR colloidal quantum dots (CQDs) have been recognized as promising material candidates owing to their broadband absorption tunability, cost‐effectiveness, and solution‐processability.^[^ ^5^ ^]^ Most eSWIR CQD photodetectors are based on Hg and Pb chalcogenides (S, Se, and Te) owing to their facile infrared (IR) absorption tunability. However, the presence of toxic heavy metals limits their commercial viability.^[^ ^6^ ^]^ Consequently, it is imperative to develop photodetectors that are non‐toxic and sensitive in the eSWIR region for commercial applications.

Silver‐chalcogenide CQDs have been proposed as non‐toxic material sources for SWIR–eSWIR photodetectors.^[^ ^7^ ^]^ Recently, a novel synthesis method for silver telluride (Ag_2_Te) CQD‐based photodetectors was proposed; however, their detection range was limited to 1.6 µm.^[^ ^8^ ^]^ Although the eSWIR photoresponse characteristics have been successfully demonstrated using Ag_2_Te CQDs at 78 and 298 K,^[^ ^9^ ^]^ significant advancements are still required in terms of dark current and response time. In particular, the photoresponse time should be improved.

In this study, we developed an eSWIR photodetector using Ag_2_Te CQDs with a nanosecond (ns) photoresponse while operating at room temperature (RT). A vertical photodiode structure was employed for operation in photovoltaic mode and to achieve a fast photoresponse. In addition to this structure, a new ligand‐exchange strategy using thiols was utilized, resulting in a reduced trap states and increased carrier mobility. Careful study of the capacitance–voltage (*C–V*) curves revealed that a favorable energy landscape tailored by the thiol ligand formed an efficient built‐in potential at the ZnO/CQD solids heterojunction, resulting in facile charge extraction. Thus, we achieved a fall time of 72 ns, which is faster than that of the previous‐best Pb‐ and Hg‐based eSWIR photodetectors.^[^ ^10^ ^]^

## 2 Results and Discussion

### 2.1 Characterization of Synthesized Ag_2_Te Colloidal Quantum Dots

**Figure 1:**
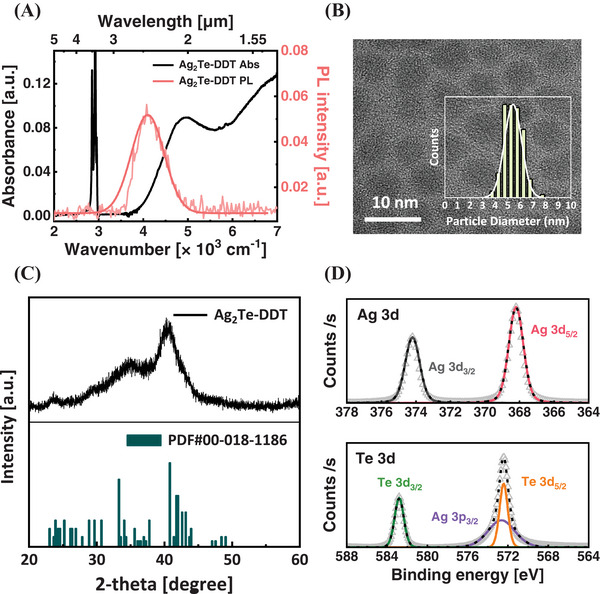
Characterization of synthesized Ag_2_Te CQDs. A) Absorbance (black) and infrared PL spectra (pale red) of the DDT‐capped Ag_2_Te CQDs. B) Transmission electron microscopy (TEM) image of DDT‐capped CQDs with a size distribution histogram (inset). C) XRD pattern of Ag_2_Te CQDs (top) and monoclinic Ag_2_Te (bottom) reference. D) XPS spectra of silver (top) and tellurium (bottom) 3d scan of Ag_2_Te CQDs.

The prior synthesis method was adjusted to produce Ag_2_Te CQDs for an eSWIR (1.7–3.0 µm) photodetector (see Experimental section).^[^ ^9^ ^]^ The optical properties of the as‐synthesized CQDs were studied using Fourier transform infrared (FT‐IR) spectroscopy and infrared photoluminescence (PL) measurements (**Figure** **1**A). A clear excitonic feature was observed at 4946 cm^−1^ (2022 nm) with a distinct PL peak at 4100 cm^−1^ (2439 nm), yielding a Stokes shift of 846 cm^−1^ (417 nm). The full‐width at half‐maximum values of the absorption and PL peaks were fitted to be ≈1040 and 870 cm^−1^, respectively. Furthermore, a uniform size distribution was observed with an average size of 5.41 ± 0.68 nm (Figure 1B), indicating the successful synthesis of eSWIR CQDs.^[^ ^8^ ^]^

We further performed X‐ray diffraction (XRD) analysis to determine the crystallographic structure of CQDs (Figure 1C). The XRD patterns revealed a monoclinic structure, indicating that the crystal structure of the CQDs aligns with that of the bulk Ag_2_Te.^[^ ^11^ ^]^ X‐ray photoelectron spectroscopy (XPS) was employed to investigate the composition of the CQDs (Figure 1D). In the narrow scan of Ag and Te 3d, peaks at 374.2 eV (3d_3/2_) and 368.2 eV (3d_5/2_) were observed for Ag atoms, while Te atoms exhibited peaks at 582.8 eV (3d_3/2_) and 572.5 eV (3d_5/2_), along with a peak at 572.7 eV corresponding to the Ag 3p_3/2_ peak in Te 3d.^[^ ^9^ ^]^ In energy‐dispersive X‐ray spectroscopy (EDS) mapping, the components of the native ligands were also observed, affirming the successful preparation of the CQDs (Figure S1, Supporting Information).

### 2.2 Surface Passivation of Ag_2_Te CQDs with Thiol Ligands

The as‐synthesized CQDs were initially capped with long alkyl chains (1‐dodecanethiol, DDT) to ensure stable dispersion in nonpolar solvents. However, to achieve efficient carrier transport in CQD solids, electronic coupling must be enhanced by exchanging the DDT ligands with other shorter ligands.^[^ ^12^ ^]^ Considering the strongly bound DDT ligands on the CQD surfaces, incoming surface ligands with comparable binding strengths are preferable. Therefore, we screened several candidates and selected three surface‐passivating ligands—1,2‐ethanedithiol (EDT), 2‐mercaptoethanol (ME), and 3‐mercaptopropionic acid (MPA).^[^ ^13^ ^]^ All three ligands have sulfur binding sites and are short, allowing facile ligand exchange. On the other hand, a halide ligand, which has a relatively lower binding affinity, did not effectively exchange the DDT ligands (Figure S2, Supporting Information). Three different thiol‐exchanged CQD solids were tested using infrared PL to predict the electronic coupling in the solid phase. Significant PL quenching was observed in the eSWIR PL spectra, confirming the promoted electronic coupling of the CQDs (Figure S3, Supporting Information).

**Figure 2:**
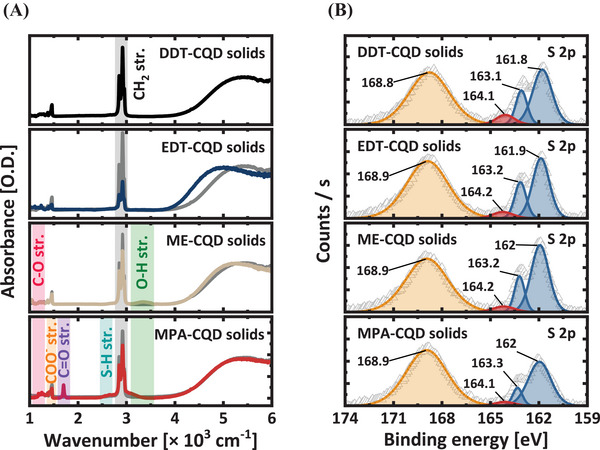
Surface passivation of Ag_2_Te CQDs with thiol ligands. A) Absorption spectra of DDT‐ (black), EDT‐ (blue), ME‐ (beige), and MPA‐CQD (red) solids. In each absorption spectrum of ligand‐exchanged CQD solids, the gray solid lines represent the absorption spectrum of DDT‐CQD solids as the baseline. B) XPS spectra of sulfur 2p scan of ligand‐exchanged CQD solids.

We further studied the FT‐IR spectra to confirm ligand exchange by identifying the ligand–CQD surface bonding (**Figure** **2**A; Table S1, Supporting Information). In DDT‐CQD solids, a strong CH_2_ peak was detected at 2852 cm^−1^ and 2922 cm^−1^, while in EDT‐CQD solids, weak CH_2_ peaks were observed, indicating that DDT ligands were replaced by EDT ligands.^[^ ^9^ ^]^ For ME‐CQD solids, additional peaks of ─OH and C─O appeared in ME‐CQD solids at 3341 cm^−1^ and 1043 cm^−1^, respectively, confirming the binding of the ME ligands onto the CQD surfaces.^[^ ^14^ ^]^ Furthermore, the presence of the ─OH peak suggests that the ─SH groups of the ME ligands are primarily coordinated to the CQD surfaces due to the lower affinity of the ─OH groups.^[^ ^14^ ^]^ Note that the negligible ─SH peak at 2560 cm^−1^ in the DDT‐, EDT‐, and ME‐CQD solids suggest that all the ligands are predominantly bound in the Ag–thiolate form.^[^ ^15^ ^]^

In MPA‐CQD solids, the C═O (1705 cm^−1^), C─O (1190 cm^−1^), and ─OH (3200 cm^−1^) peaks indicate that the thiol end of the MPA ligands is bound to the CQD surfaces.^[^ ^16^ ^]^ Weak ─SH (2578 cm^−1^) and COO^−^ (1464 cm^−1^) peaks suggest the binding formation of Ag–thiolate due to the favorable affinity of ─SH groups toward Ag atoms,^[^ ^15^ ^]^ correlating with density functional theory (DFT) calculations (Figure S4, Supporting Information). Subsequently, XPS analysis was performed to clarify the ligand exchange. Analogous Ag and Te 3d spectra were observed in three different CQD solids (Figure S5, Supporting Information). The C 1s spectra were studied to identify the functional groups and showed bonding characteristics that were identical to the FT‐IR results. Furthermore, while the native DDT‐CQD solids exhibited substantial oxidation states from the O 1s spectra, negligible surface oxidations were observed after ligand exchange, suggesting successful surface reconstruction (Figure S6, Supporting Information).

Off‐stoichiometry remained nearly constant after ligand exchange (Ag/Te ≈2.5), indicating that thiol ligands replace the DDT ligands, rather than the Ag–DDT complex (Figure S7, Supporting Information). In contrast to CQDs with covalent surfaces, which exhibit nearly balanced stoichiometry after ligand exchange, those with ionic surfaces show no significant changes in their stoichiometry.^[^ ^17^ ^]^ This allows us to conclude that Ag_2_Te CQDs exhibit an ionic bonding nature, typically observed in II‐VI and IV‐VI CQDs.^[^ ^18^ ^]^

To elucidate the efficacy of the ligand exchange, we analyzed the narrow scan of S 2p spectra (Figure 2B). The 168.9 eV peak at the higher binding energy corresponds to the sulfate of Ag─SO_4_ or Te 4s;^[^ ^19^ ^]^ however, negligible oxidized species for ligand‐exchanged CQD solids (Figure S6, Supporting Information) suggest that the 168.9 eV peak corresponds to Te 4s rather than sulfate. The S 2p peak was deconvoluted into three main peaks for further study. Peaks at 162 eV (2p_1/2_) and 163.2 eV (2p_3/2_) exhibit a 1.2 eV splitting due to spin‐orbit coupling, while the peak at 164.1 eV corresponds to unbound thiolate.^[^ ^20^ ^]^ We observed that the area ratio of unbound thiolate to S 2p decreased after ligand exchange (Table S3, Supporting Information). Specifically, EDT‐, ME‐, and MPA‐CQD solids exhibited area ratios of 0.089, 0.079, and 0.066, respectively, indicating that the MPA‐CQD solids exhibit the most efficient ligand exchange with DDT ligands. This result agrees with the FT‐IR spectra results shown in Figure 2A in that the MPA‐CQD solids exhibit a red‐shift that is smaller by 60 cm^−1^ (Table S2, Supporting Information). Because a larger red shift of the absorption peak indicates a higher degree of energetic disorder after solid‐state ligand exchange,^[^ ^21^ ^]^ it can be inferred that MPA is the most effective ligand for passivating the Ag_2_Te CQD surfaces.

Given that highly passivated CQDs enhance electrical properties,^[^ ^22^ ^]^ we investigated the electrical characteristics of CQD solids. We employed the space‐charge‐limited current (SCLC) method to determine the trap densities (*n*_t_) and hole mobilities (*µ*_h_), as shown in Figure S8 and Note S1 (Supporting Information). Indeed, MPA‐CQD solids exhibited the lowest *n*_t_ of 9 × 10^15^ cm^−3^ and the highest *µ*_h_ of 2.8 × 10^−2^ cm^−2^ V^−1^ s^−1^, which is approximately two times lower in terms of *n*_t_ and seven times higher in terms of *µ*_h_ compared to EDT‐CQD solids (Table S4, Supporting Information). We attributed the reduced *n*_t_ in MPA‐CQD solids to the more efficient surface passivation compared to ME‐ and EDT‐CQD solids. Note that the improved electrical properties were not attributed to the morphology of the CQD solids, as the average roughness exhibited negligible differences under each condition (Figure S9, Supporting Information). The SCLC results corroborate with the previous spectroscopic analyses (Figure 2), indicating that an efficient passivation of the MPA ligand enhances the conductivity of CQD solids.

### 2.3 Energy Band Alignment of Ag_2_Te CQD Solids

**Figure 3:**
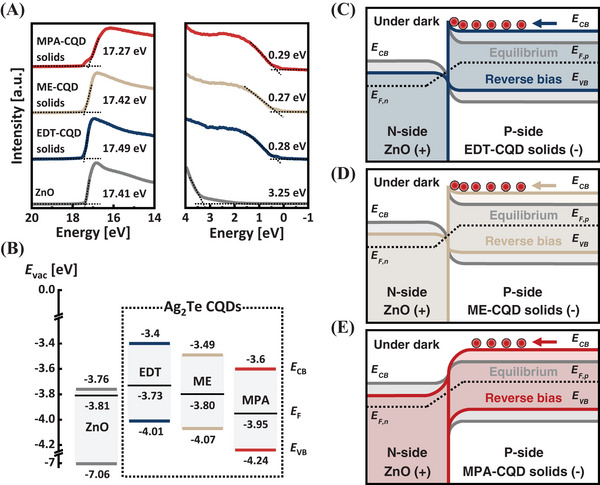
Energy band alignment of ligand‐exchanged Ag_2_Te CQD solids. A) Binding energy spectra measured by UPS. B) Energy‐level diagrams for ZnO (gray), EDT‐ (blue), ME‐ (beige), and MPA‐CQD (red) solids from UPS measurements. *E*_vac_, *E*_F_, *E*_CB_, and *E*_VB_ indicate the vacuum level, Fermi level, conduction band edge, and valence band edge, respectively. C–E) Schematic energy level diagrams at the ZnO/CQD solids junction at equilibrium (gray) and under reverse bias (colored) in the dark. *E*_CB_, *E*_VB_, *E*_F,p_, *E*_F,n_ represent the conduction band edge, valence band edge, quasi‐Fermi level of p‐type, and quasi‐Fermi level of n‐type, respectively. Negative charges (red circles) denote thermally generated minority carriers (electrons).

Fine‐tuning of the energy levels is imperative for enhanced carrier extraction and lower dark current, especially with low‐bandgap materials.^[^ ^23^ ^]^ In particular, this modulates the internal electric field, which is crucial for operating photodetectors in photovoltaic mode.^[^ ^24^ ^]^ To identify the energy level, the valence band edge (*E*_VB_), and Fermi level (*E*_F_) for each condition were analyzed using ultraviolet photoelectron spectroscopy (UPS) (**Figure** **3**A). The cut‐off wavelengths from the FT‐IR spectra were employed to determine the conduction band edge (*E*_CB_) for each condition, and the resultant energy levels are illustrated in Figure 3B.

All the CQD solids exhibited a marginal p‐type property, consistent with the results of previous studies,^[^ ^25^ ^]^ as confirmed by field‐effect transistor (FET) measurements (Figure S10, Supporting Information); however, their energy levels varied depending on the surface ligands.^[^ ^26^ ^]^ Compared to the ZnO electron transport layer (ETL), the EDT‐ and ME‐CQD solids exhibited a slightly shallower *E*_F_, while the MPA‐CQD solids possessed a 0.14 eV deeper *E*_F_. The overall energy level diagrams of the ZnO/CQD solids junctions are shown in Figure 3C–E.

For both the ZnO/EDT‐ and ZnO/ME‐CQD solids junctions, under dark conditions at equilibrium, the energy levels of ZnO bend downward toward the junction interface, while the energy levels of the CQD solids bend upward (Figure 3C,D). Under a reverse bias, ZnO has deeper energy levels, while the EDT‐ and ME‐CQD solids have shallower energy levels, leading to the accumulation of thermally generated minority carriers (electrons) at the junction interface. However, the ZnO/MPA‐CQD solids junction conforms to the conventional p–n junction properties due to the deeper *E*_F_ of the MPA‐CQD solids (Figure 3E).^[^ ^27^ ^]^ Consequently, minority carriers are not accumulated at the junction interface under reverse bias.

To validate the energy level diagrams, we conducted capacitance–voltage (*C*–*V*) measurements under dark conditions (Figure S11, Supporting Information). All three devices showed a decrease in capacitance (*C*_P_) due to depletion under reverse bias.^[^ ^27^ ^]^ While the EDT‐ and ME‐CQD devices exhibited an increase in *C*_P_ (i.e., inversion) after depletion, the MPA‐CQD devices did not exhibit inversion behavior, indicating a typical p‐n junction that yields a sufficient depletion width (Figure S12, Table S5, and Note S2, Supporting Information). Thus, the MPA‐CQD solids exhibit the most favorable energy band structure with ZnO.

### 2.4 Device Performance of Ag_2_Te CQD eSWIR Photodetectors

Various types of eSWIR photodetectors based on non‐toxic silver chalcogenide Ag_2_X (X = S, Se, and Te) nanocrystals have been proposed.^[^ ^8^, ^28^ ^]^ In this study, we introduced a photodiode architecture to take advantage of the fast photoresponse time and low dark current of the vertical structure.^[^ ^29^ ^]^

**Figure 4:**
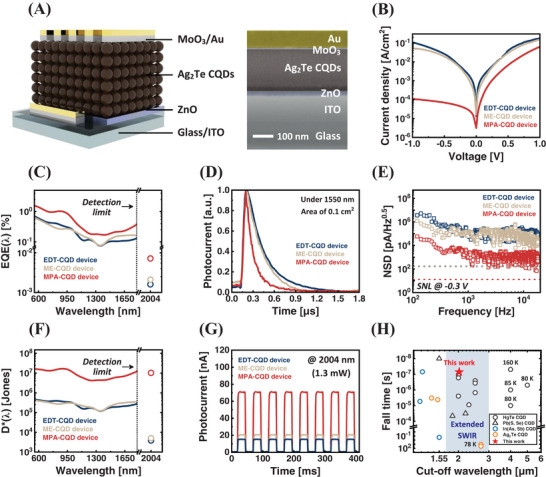
Device performance of Ag_2_Te CQD eSWIR photodetectors. A) Device architecture and a cross‐sectional SEM image (scale bar: 100 nm). B) Current density–voltage (*J*–*V*) curves of EDT‐ (blue), ME‐ (beige), and MPA‐CQD (red) devices under dark conditions. C) EQE(λ) spectra of EDT‐ (blue), ME‐ (beige), and MPA‐CQD (red) devices. The dotted line indicates the limit of the instrument (≈1.8 µm). The EQEs below this detection limit were measured directly, while a 2004 nm laser was utilized beyond 1.8 µm. D) On‐off characteristics under short‐circuit conditions with a 2004 nm irradiation of EDT‐ (blue), ME‐ (beige), and MPA‐CQD (red) devices. E) Frequency‐dependent noise spectral density (NSD), and F) *D* ^*^(λ) spectra of EDT‐ (blue), ME‐ (beige), and MPA‐CQD (red) devices. G) Transient photoresponse for the EDT‐ (blue), ME‐ (beige), and MPA‐CQD (red) devices under 1550 nm pulsed irradiation and short‐circuit conditions. H) Figure of merit (FOM) consisting of cut‐off wavelength versus fall time for previously reported CQD‐based photodetectors. The shaded area indicates the eSWIR band (1.7–3.0 µm).

A schematic of the eSWIR Ag_2_Te CQD photodiode, scanned by a cross‐sectional focused‐ion beam, is shown in **Figure** **4**A. The overall device structure includes the following layers: indium tin oxide (ITO) as a transparent electrode (≈70 nm), ZnO as an ETL (≈30 nm), a CQD‐absorbing layer (≈236 nm), MoO_3_ as a hole transport layer (≈10 nm), and a gold‐top electrode (≈100 nm). Note that the thickness of the CQD‐absorbing layer was characterized using a surface profiler, and CQDs with a size of 5.41 nm (1st excitonic peak at 2022 nm) were used for eSWIR photodiodes.

A current density–voltage (*J–V*) curve was examined under dark conditions to verify the diode characteristics (Figure 4B). The MPA‐CQD devices exhibited a significantly suppressed dark current density (*J*_d_) of 1.1 × 10^−4^ A cm^−2^ at −1 V, which is two orders of magnitude lower than that of the EDT‐ and ME‐CQD devices, yielding a rectification ratio^[^ ^17^ ^]^ of 920 (Figure S13 and Table S6, Supporting Information). As shown in Figure 3, the deeper *E*_F_ of the MPA‐CQD solids leads to the suppression of minority carrier diffusion and thereby reduces the dark current. Furthermore, the efficient passivation of the MPA ligands also decreased trap‐assisted recombination, thus reducing the dark current.^[^ ^30^ ^]^ Notably, the EDT‐ and ME‐CQD device exhibited a higher *J*_d_ at +1 V due to the facile transport of majority carriers at the junction (Figure S14, Supporting Information).

We also observed the *J*–*V* curves under illumination to explore the relationship with the internal electric field (Figure S15, Supporting Information). While the EDT‐ and ME‐CQD devices displayed negligible open‐circuit voltage (*V*_OC_), the MPA‐CQD devices exhibited a *V*_OC_ of 0.02 V under both types of irradiation. The deeper *E*_F_ and the lower trap states in the MPA‐CQD solids contributed to the lower *V*_OC_ losses.^[^ ^31^ ^]^ However, the small *V*_OC_ (0.02 V) compared to the built‐in potential (*V*_bi_) of 0.18 V indicates that several loss mechanisms still exist in low bandgap MPA‐CQD devices, such as interfacial and non‐radiative recombination (Figure S11, Supporting Information).^[^ ^32^ ^]^

Next, the spectral external quantum efficiencies (EQEs) were examined. As the 1st excitonic peak of CQDs used in eSWIR photodiodes exceeds the detection limitations (≈1.8 µm), a collimated 2004 nm laser was employed to study the actual performance of the photodetectors beyond 2 µm (Figure 4C,D). The MPA‐CQD devices exhibited the highest EQE of 0.21% at 1550 nm and 0.0072% at 2004 nm, respectively. In conjunction with the R(λ) spectra in Figure S16 (Supporting Information), the MPA‐CQD devices demonstrated improved charge extraction at both wavelengths.

Subsequently, noise current (*i*_n_) measurements were performed to extract the specific detectivity (*D* ^*^), with the shot‐noise limit (*SNL*) set to −0.3 V, as derived from *J*–*V* curves (Figure 4E; Note S3, Supporting Information). All the devices exhibited frequency‐dependent flicker behavior, with *i*_n_ decreasing as the frequency increased.^[^ ^22b^ ^]^ At 10 kHz, the *i*_n_ of the EDT‐ and ME‐CQD devices were similar, measuring 2.1 × 10^−8^ A/Hz^0.5^, which is approximately two orders of magnitude higher than their *SNL*, while the MPA‐CQD devices showed an *i*_n_ value of 1.37 × 10^−10^ A/Hz^0.5^, approximately one order of magnitude higher than that of the *SNL*. Consequently, we calculated *D* ^*^ using the following equation and presented *D* ^*^ as a function of wavelength in Figure 4F.

(1) $$D^{*}=\frac{{\left(A\times \Delta f\right)}^{1/2}\times R}{i_{n}}$$

where *R* represents the responsivity, *i*_n_ is the noise current (at 10 kHz), *A* is the device area (0.1 cm^2^), and Δ*f* is the electrical bandwidth (set to 1 Hz). The *D* ^*^ of the EDT‐, ME‐, and MPA‐CQD devices were determined to be 2.0 × 10^4^, 2.9 × 10^4^, and 6.1 × 10^6^ Jones at 1550 nm, and 3.8 × 10^3^, 5.0 × 10^3^, and 1.0 × 10^7^ Jones at 2004 nm, respectively, at room temperature. Note that the *i*_n_ at 0 V was utilized to calculate the *D* ^*^ at 2004 nm due to the low signal‐to‐noise ratio under biased conditions (Figure S17, Supporting Information). The best *D* ^*^ was achieved for the MPA‐CQD devices at both wavelengths. The higher performance of MPA‐CQD devices originates from the superior electrical properties and favorable energy band alignment tailored by MPA ligands. Note that the low *D* ^*^ in the eSWIR wavelength originates from the low absorption coefficient of Ag_2_Te CQDs, suggesting that the thicker CQD solids would be the next challenge to improving the performance (Figure S18, Supporting Information). Furthermore, achieving high performance in eSWIR photodetection is challenging at 298 K due to the small bandgap.^[^ ^1^, ^9^ ^]^ To this end, further studies are needed to suppress thermally generated carriers and extend minority carrier lifetimes.^[^ ^33^ ^]^

Considering potential infrared applications, long‐range communications, and object recognition such as LiDAR, fast photoresponse under a pulsed laser is important. We studied the transient photocurrent (TPC) using 1550 nm pulsed irradiation. The representative TPC results for all the devices are shown in Figure 4G. The fall times (90% to 10%) for EDT‐, ME‐ and MPA‐CQD devices were 363, 235, and 200 ns, respectively. Note that we attribute the discrepancy in response time under two light sources (1550 and 2004 nm) to different irradiation methodologies and a longer extraction path resulting from the absorption depth.^[^ ^34^ ^]^ A device‐area‐dependent TPC was also performed to explore the impact of device geometry. As the device area decreased from 0.1 to 0.009 cm^2^, the fall times of all the devices decreased (Figure S19 and Table S7, Supporting Information). In particular, the MPA‐CQD devices demonstrated the fastest fall time of 72 ns at 0.009 cm^2^. This implies that the improved carrier mobility via efficient passivation by MPA ligands, and the generated *V*_bi_ with MPA‐CQD solids, leads to enhancements in both the drift and diffusion of charge carriers.^[^ ^35^ ^]^ A small‐area device shows undershooting during TPC measurement, presumably due to the increased impact of *n*_t_.

A light‐intensity‐dependent measurement (i.e., Linear dynamic range, *LDR*) and a −3 dB bandwidth (*f*_−3 dB_) measurement were performed to improve the reliability of the photodetectors. The EDT‐, ME‐, and MPA‐CQD devices yielded *LDR*s of 44.9, 32.7, and 78.8 dB, respectively (Figure S20, Supporting Information). In addition to LDR, both the EDT‐ and ME‐CQD devices demonstrated a *f*_−3 dB_ of 100 kHz, whereas the MPA‐CQD devices exhibited a *f*_−3 dB_ exceeding 102 kHz, which is the measurement limit (Figure S21, Supporting Information). Based on the obtained results, we investigated recent research into CQD photodetectors across a detection range of 1.1–6 µm, presenting the cut‐off wavelength versus fall time as a Figure of merit (Figure 4H; Table S8, Supporting Information). In this work, we achieved fall time that was faster than any prior CQD‐based eSWIR photodetectors, even those including toxic elements (Figure S22, Supporting Information). The origin of the fast response of the MPA‐treated Ag_2_Te CQD device is improved electrical properties, and we speculate that the intrinsically low effective mass of Ag_2_Te enables a further increase in carrier mobility, thereby boosting the response time (Note S4, Supporting Information).^[^ ^36^ ^]^

## 3 Conclusion

In this study, we demonstrated an eSWIR Ag_2_Te CQD photodetector with a fast photoresponse. The optimum ligand‐exchange strategy for non‐toxic Ag_2_Te CQDs effectively reduced the trap density of Ag_2_Te CQD solids and significantly enhanced the hole mobility, yielding a fast eSWIR photodiode. Furthermore, an efficient heterojunction was finely tuned via surface modification, resulting in efficient charge‐carrier extraction and effective dark current suppression. Given the intricate crystallographic structure, a more in‐depth exploration of multiple facets would improve the performance of Ag_2_X (X = S, Se, and Te) CQD‐based eSWIR photodetectors.^[^ ^30^, ^37^ ^]^ Additionally, we anticipate that employing CMOS‐compatible alternatives (e.g., W, Cu) instead of Au electrodes,^[^ ^38^ ^]^ adopting non‐electrode structures,^[^ ^39^ ^]^ or integrating Te‐anchorable IR cavity structures^[^ ^40^ ^]^ would pave the way for the development of CMOS‐applicable Ag_2_Te CQD imagers.

## 4 Experimental Section

### Materials

The study purchased silver acetate (AgAc, 99%), 1‐dodecanethiol (DDT) (≥98%), trioctylphosphine (TOP, tech. grad. 90%), zinc acetate dihydrate (≥98%), ethanolamine (≥98%), 2‐methoxyethanol (anhydrous), and 1,2‐ethanedithiol (EDT) (98%) from Sigma Aldrich; tellurium powder (Te, 200 mesh, 99.5%) from Alfa Aesar, and *n*‐octane (≥98%); and 3‐mercaptopropionic acid (MPA) (99%) from Thermofisher. In addition, 2‐mercaptoethanol (ME, extra pure) was procured, chloroform, and isopropyl alcohol (IPA, HPLC grade) from Daejung and ethyl acetate (EA, HPLC grade) from Duksan.

### Synthesis of Ag_2_Te Colloidal Quantum Dots

Ag_2_Te CQDs were prepared as previously described.^[^ ^9^ ^]^ AgAc (8 mmol) and DDT (80 mL) were placed in a 250 mL three‐neck round‐bottom flask and purged with Ar gas for 30 min. When the temperature was raised to 90 °C, the solution turned a clear yellow color; 4 mL of 1‐M TOP‐Te was quickly injected at this time. After 10 min of reaction, the product solution containing chloroform and IPA was centrifuged to remove residual byproducts.

### Preparation of ZnO Sol–Gel

To establish the electron transport layer, the ZnO sol–gel was prepared by stirring 1 g of zinc acetate dihydrate with 0.277 mL of ethanolamine and 10 mL of anhydrous 2‐methoxyethanol overnight.

### Device Fabrication

Before the fabrication of the devices, the ITO glass pieces underwent a meticulous three‐step cleaning process. Initially, they were sonicated in deionized water with a few drops of detergent for 15 min, followed by a 15‐min wash in acetone and a subsequent 15‐min immersion in isopropyl alcohol. The ITO glass pieces were then dried in a vacuum oven for 20 min. Subsequently, the dried ITO glass pieces underwent a 15‐min UV ozone treatment, followed by spin coating with the prepared ZnO sol–gel at 3000 rpm for 30 s, and finally, annealing at 235 °C for 30 min in ambient air. To achieve surface reconstruction, the as‐synthesized DDT‐capped CQDs were dispersed in *n*‐octane at a concentration of 65 mg mL^−1^. In addition, solutions of 0.5 vol% EDT, ME, and MPA in EA were prepared as surface modifiers. The layer‐by‐layer (LBL) technique was performed using a solid‐state ligand‐exchange method under ambient air conditions. Approximately 50 µL of a 65 mg mL^−1^ CQD solution was dispensed onto the ZnO layer, and spin‐coated at 2500 rpm for 8 s. The thiolate ligand precursor was subsequently evenly applied and spin‐coated directly at 2500 rpm for 8 s without any soaking. Subsequently, the structure was rinsed three times with pure EA to eliminate residual ligands. The entire sequence has been denoted as one cycle, and the LBL process was repeated until the desired thickness was achieved. For the hole transport layer, MoO_3_ with a thickness of 10 nm was deposited via thermal evaporation; a top Au electrode (100 nm) was deposited using the same technique.

### Characterization

*FT‐IR*: All FT‐IR absorption spectra were measured at 298 K using a Nicolet iS10 FT‐IR spectrometer with a resolution of 0.482 cm^−1^.

*PL*: A homemade mid‐IR emission spectrometer was used to measure the PL of the untreated and ligand‐exchanged Ag_2_Te CQDs. A 532‐nm Nd:YAG pulsed laser (EKSPLA, NL300) with a width of 5 ns and repetition rate of 10 Hz was employed as the excitation source. A HgCdTe (MCT) detector was used to collect mid‐IR photons from the samples.

*TEM*: The morphology and size of the Ag_2_Te CQDs were investigated using a JEM‐2100F microscope. The TEM instrument was operated at an acceleration voltage of 200 kV using a ZrO/W(100) Schottky electron source.

*XRD*: A D8 Discover + (Bruker AXS GmbH) X‐ray diffractometer (XRD) with a 6 kW high‐power X‐ray source was utilized to identify the crystal structure of the Ag_2_Te CQDs. The diffractometer had a focal brightness of 6 kW mm^−2^, and the XRD pattern was collected from 20° to 60° in 0.01° increments.

*EDS*: For the elemental analysis, EDS mapping was conducted on CQD solids deposited on a cleaned ITO substrate using high‐resolution FE‐SEM (Hitachi S‐4800).

*Cross‐Sectional Focused Ion Beam*: The cross‐sections of the Ag_2_Te photodiodes were obtained using a focused ion beam instrument (Thermofisher, Helios 5 UC).

*DFT Calculations*: The binding energies of the ligands were obtained by DFT calculations at the B3LYP/LanL2DZ level using the Gaussian 16 W program package.^[^ ^41^ ^]^

*AFM*: For the AFM measurements, the exchanged CQD films were deposited onto cleaned glass/ITO substrates. AFM measurements were conducted using an NX20 instrument (Park Systems) operating in tapping mode at a scan rate of 1 Hz. The AFM topographic images of the films, which covered an area of 20 × 20 µm, were utilized to quantify the root‐mean‐square and arithmetic average roughness of the films.

*XPS and UPS*: The XPS measurement was performed under vacuum conditions using a Thermofisher Scientific Nexsa with monochromated Al Kα sources. For the XPS spectra, the energy calibration was based on the value of C 1s at 284.8 eV. Energy diagrams were obtained from the UPS measurements at a scan rate of 0.1 eV and a pass energy of 50 eV. Ag and Au electrodes were employed as reference electrodes for the calibration. For the UPS measurements, a He‐I lamp with a photon energy of 21.22 eV was employed, and the Fermi level (*E*_F_) was determined by utilizing the equation provided below.

(2) $$E_{F}=hv-\left|E_{cut-off}\right|$$

where *hν* is the photon energy of the He‐I light source, 21.22 eV, and *E*_cut‐off_ is the energy of the fitted secondary cut‐off. The *E*_VB_ was subtracted from the *E*_F_ by the primary cut‐off energy, and the *E*_CB_ was extracted from the converted bandgap (1240 nm/*λ*_cut‐off_). The *λ*_cut‐off_ is obtained from the corresponding FT‐IR spectra.

*Absorption coefficient*: To determine the absorption coefficient (α) of the CQD solids, a UV—vis–NIR spectrophotometer (Jasco, V‐770) with an integrating sphere was employed. First, CQD solids were prepared on ITO glass pieces, and the transmittance was measured. The film thickness was characterized using a surface profiler (Dektak‐XT, Bruker), and the value of α was subsequently calculated using the provided equation.

(3) $$\alpha \left[cm^{-1}\right]=\frac{2.303\times log_{10}\left(T[\%]/100\right)}{d[cm]}$$

where *T* is the measured transmittance of the film and *d* is the thickness of the film.

*C–V*: The *C*–*V* curves were measured at 298 K using Ivium compactstat.h (IVIUM) with a frequency of 10 kHz, and the bias was scanned from +1.0 to −1.0 V.

*FET*: For FET measurements, heavily n‐doped Si wafers (n^++^, <0.005 ohm), coated with a thermally grown SiO_2_ layer (200 nm) were utilized as the substrate. The substrates underwent cleaning procedures identical to those employed for the ITO glass pieces, followed by treatment with UV ozone. Subsequently, ligand‐exchanged CQD solids were deposited onto each substrate using the LBL technique, and a top Au electrode was thermally evaporated to serve as the source, drain, and gate electrodes. To ascertain the doping types, the gate voltage (V_GS_) was swept from +15 to −60 V with a constant drain voltage (V_DS_) of +10 V.

*J–V*: The *J*–*V* curves of the photodetectors were measured using a Keithley 2401 source meter at 298 K in the dark. For *J*–*V* curves under light conditions, a 1550 nm CW laser (MDL‐III−1550‐100 mW diode laser) equipped was utilized with a collimator (FOC‐01‐A), and a 940‐nm pulsed laser (MDL‐NS‐940‐100 mW) with a pulse width of 500 ns and a repetition rate of 1 MHz. The *J*–*V* curves were swept from −1.0 to 1.0 V in 0.02 V intervals, with a 50 ms delay at each step.

*EQE*: The EQE spectra were acquired using the QuantX‐300 measurement system (Newport). Monochromatic white light from a 400‐W Xenon lamp, modulated at a frequency of 220 Hz, was directed onto the device, and spectral responses were derived from the measured photocurrent. The EQE spectrum was utilized to calculate the responsivity of the photodetector.

(4) $$R=EQE\times \frac{\lambda \;\left[nm\right]}{1240\;\left[nm\right]}$$

where *R*, *EQE*, and *λ* represent the responsivity, external quantum efficiency, and wavelength, respectively. EQE measurements were performed under a bias of −0.3 V.

*Noise Current Measurement*: All device systems are wrapped in aluminum foil before measuring noise current minimize the influence of external electric fields.^[^ ^42^ ^]^ The noise voltages were amplified using a low‐noise current amplifier (SR570, Stanford Research Systems). The signals were recorded with an oscilloscope (DSO7054A, Tektronix) using fast Fourier transform (FFT), with a resolution bandwidth of 20 Hz. Note that all noise–current measurements were performed at 298 K under −0.3 V and short‐circuit conditions, respectively, in dark conditions.

*On–Off Characteristics*: The on–off characteristics were verified using 2004 nm continuous‐wave (CW) lasers (EP2004 and LM14S2) modulated at 20 Hz with an optical chopper. To enhance the precision, a collimator (TC25APC‐2000) was employed. The photovoltage was amplified via an SR570 transimpedance preamplifier (Stanford Research Systems) under short‐circuit conditions, and the signals were recorded with an oscilloscope (DSO7054A, Tektronix). The photocurrent was determined by converting the amplitude of the amplified signals. Due to the low signal‐to‐noise ratio under biased conditions, the on–off characteristics were measured without applying a bias.

*TPC*: For the TPC measurements, a 1550 nm pulsed laser (VFLS‐1550‐M‐PL‐30‐10 for DTS) was used with a pulse width of 50 ns and repetition rate of 10 kHz. All the photoresponse signals were recorded with a 500‐MHz oscilloscope (DSO7054A, Tektronix) at an input impedance of 50 Ω.

*LDR*: In LDR measurements, photodetectors with an area of 0.1 cm^2^ were utilized. A 1550 nm CW laser was employed, and anti‐reflective (AR)‐coated absorptive neutral density filters (Thorlabs) were utilized to accurately regulate the light intensity. The LDR was calculated through linear fitting using the following equation.

(5) $$LDR=20\times log_{10}\left(I_{photo,\;max}/I_{photo,\;min}\right)$$

where *I*_photo, max_ is the measured maximum photocurrent linearly correlated with increasing light intensity, and *I*_photo, min_ is the measured minimum photocurrent discernible from the dark current.

−*3 dB bandwidth*: To determine the −3 dB bandwidth (*f*_−3 dB_), the study employed an active area of 0.1 cm^2^ and utilized an SR830 lock‐in amplifier and an oscilloscope under 1550 nm CW laser irradiation. The resulting signals were recorded and analyzed using the oscilloscope, and the *f*_−3 dB_ was calculated using the following equation.

(6) $$\;f_{-3dB}=10\times \log_{10}\left(\frac{V_{i}}{V_{f}}\right)$$

where *V*_i_ is the initial voltage (in this case, the frequency is set to 100 Hz), and *V*_f_ is the final voltage at the frequency corresponding to the *f*_−3 dB_. Note that, for frequencies below 100 Hz, no voltage change was observed in any of the devices with variations in the frequency. Therefore, all *f*_−3 dB_ measurements were initiated from 100 Hz under ambient air conditions at 298 K.

*SCLC Methods*: An overall hole‐only device was constructed with a multilayered structure consisting of ITO/MoO_3_/Ag_2_Te CQDs/MoO_3_/Au, with MoO_3_ used as the electron‐blocking layer. For the SCLC measurements, an active area of 0.1 cm^2^ was used, and the *J–V* curves were swept from 0.001 to 1 V in 0.01 V intervals under dark conditions.

## Conflict of Interest

The authors declare no conflict of interest.

## Author Contributions

Y.A. and S.Y.E. equally contributed to this work. Y.A. and S.Y.E. contributed equally to this study by conceptualizing and developing it. Y.A. fabricated the devices, characterized their performance, and analyzed their electronic characteristics. S.Y.E. synthesized the Ag_2_Te CQDs and conducted the DFT calculations. G.K. performed the optical analysis of the Ag_2_Te CQDs, J.H.L. conducted the PL measurements of the CQD solids, and B.K., D.K., M.‐J.S., and M.Y. contributed to the optical and electrical analyses. Y.J., B.S.K., and Y.J.C. performed the *C*–*V* and FET measurements and engaged in the subsequent discussions. K.S.J. and S.‐W.B. oversaw and supervised the study. All the authors actively participated in discussions regarding the results and offered valuable comments on the manuscript.

## Supporting information



Supporting Information

## Data Availability

The data that support the findings of this study are available from the corresponding author upon reasonable request.

## References

[1] a) M. Chen , H. Lu , N. M. Abdelazim , Y. Zhu , Z. Wang , W. Ren , S. V. Kershaw , A. L. Rogach , N. Zhao , ACS Nano 2017, 11, 5614; 28525710 10.1021/acsnano.7b00972

[2] a) S. Lin , J. Chang , J. Sun , P. Xu , Front. Phys. 2022, 10, 853966;

[3] a) C. Li , Y. Zhang , K. Wang , Y. Gu , H. Li , Y. Li , Infrared Phys. Technol. 2010, 53, 173;

[4] Y. Tian , H. Luo , M. Chen , C. Li , S. V. Kershaw , R. Zhang , A. L. Rogach , Nanoscale 2023, 15, 6476. 36960839 10.1039/d2nr07309a

[5] S. Keuleyan , E. Lhuillier , P. Guyot‐Sionnest , J. Am. Chem. Soc. 2011, 133, 16422. 21942339 10.1021/ja2079509

[6] a) D. M. Balazs , M. A. Loi , Adv. Mater. 2018, 30, 1800082; 10.1002/adma.201802265 30069938

[7] a) R. Bera , D. Choi , Y. S. Jung , H. Song , K. S. Jeong , J. Phys. Chem. Lett. 2022, 13, 6138; 35759614 10.1021/acs.jpclett.2c01179

[8] Y. Wang , L. Peng , J. Schreier , Y. Bi , A. Black , A. Malla , S. Goossens , G. Konstantatos , Nat. Photonics 2024, 18, 236.

[9] G. Kim , D. Choi , S. Y. Eom , H. Song , K. S. Jeong , Nano Lett. 2021, 21, 8073. 34524828 10.1021/acs.nanolett.1c02407

[10] a) T. Zhu , Y. Yang , L. Zheng , L. Liu , M. L. Becker , X. Gong , Adv. Funct. Mater. 2020, 30, 1909487;

[11] T.‐T. Yeh , W. H. Lin , W.‐Y. Tzeng , P. H. Le , C.‐W. Luo , T. I. Milenov , J. Alloys Compd. 2017, 725, 433.

[12] a) C. R. Kagan , C. B. Murray , Nat. Nanotechnol. 2015, 10, 1013; 26551016 10.1038/nnano.2015.247

[13] a) M. M. Ackerman , X. Tang , P. Guyot‐Sionnest , ACS Nano 2018, 12, 7264; 29975502 10.1021/acsnano.8b03425

[14] C. Wang , Q. Wu , Y. Wang , Z. Wang , H. Li , X. Li , X. Chen , C. Wang , Y. Liu , X. Zhang , Adv. Funct. Mater. 2024, 34, 2315365.

[15] S. Y. Bae , J. Yang , J. T. Oh , C. B. Lee , H. Song , B. R. Lee , H. M. Jin , K. Kim , J. P. Hong , Y. Kim , Chem. Eng. J. 2023, 474, 145674.

[16] a) S. Veerasingam , R. Venkatachalapathy , Infrared Phys. Technol. 2014, 66, 136;

[17] M.‐J. Choi , L. K. Sagar , B. Sun , M. Biondi , S. Lee , A. M. Najjariyan , L. Levina , F. P. García de Arquer , E. H. Sargent , Nano Lett. 2021, 21, 6057. 34250796 10.1021/acs.nanolett.1c01286

[18] a) J.‐H. Ko , D. Yoo , Y.‐H. Kim , Chem. Commun. 2017, 53, 388; 10.1039/c6cc07933d 27942624

[19] R. Manchanda , V. Sharma , V. S. , Chanchal , A. Goyal , V. Srivastava , A. K. Saini , N. Singh , R. S. Saxena , R. Raman , at The Physics of Semiconductor Devices: Proceedings of IWPSD 2017 , Springer International Publishing, 2019, pp.453–460.

[20] D. G. Castner , K. Hinds , D. W. Grainger , Langmuir 1996, 12, 5083.

[21] a) P. Guyot‐Sionnest , J. Phys. Chem. Lett. 2012, 3, 1169; 26288053 10.1021/jz300048y

[22] a) D. Zhitomirsky , O. Voznyy , L. Levina , S. Hoogland , K. W. Kemp , A. H. Ip , S. M. Thon , E. H. Sargent , Nat. Commun. 2014, 5, 3803; 24801435 10.1038/ncomms4803

[23] a) S. H. Kim , D. Lee , S. Moon , J. H. Choi , D. Kim , J. Kim , S. W. Baek , Adv. Funct. Mater. 2023, 33, 2303778;

[24] M. Vafaie , J. Z. Fan , A. M. Najarian , O. Ouellette , L. K. Sagar , K. Bertens , B. Sun , F. P. G. de Arquer , E. H. Sargent , Matter 2021, 4, 1042.

[25] P. Zhao , T. Qin , G. Mu , S. Zhang , Y. Luo , M. Chen , X. Tang , J. Mater. Chem. C 2023, 11, 2842.

[26] B. Kim , S. W. Baek , C. Kim , J. Kim , J. Y. Lee , Adv. Energy Mater. 2022, 12, 2102689.

[27] B. K. Jung , H. Yoo , B. Seo , H. J. Choi , Y. K. Choi , T. H. Kim , N. Oh , S. Y. Kim , S. Kim , Y. Lee , ACS Energy Lett. 2024, 9, 504.

[28] a) H. Roshan , M. H. Sheikhi , A. Mirzaei , T. Kaewmaraya , T. Hussain , R. Brescia , J. Alloys Compd. 2023, 939, 168754;

[29] K. Xu , W. Zhou , Z. Ning , Small 2020, 16, 2003397. 10.1002/smll.202003397 33140560

[30] Y. Xia , W. Chen , P. Zhang , S. Liu , K. Wang , X. Yang , H. Tang , L. Lian , J. He , X. Liu , Adv. Funct. Mater. 2020, 30, 2000594.

[31] a) J. Wang , EcoMat 2022, 4, e12263;

[32] J. Liu , K. Xian , L. Ye , Z. Zhou , Adv. Mater. 2021, 33, 2008115. 10.1002/adma.202008115 34085736

[33] R. Ollearo , J. Wang , M. J. Dyson , C. H. Weijtens , M. Fattori , B. T. Van Gorkom , A. J. Van Breemen , S. C. Meskers , R. A. Janssen , G. H. Gelinck , Nat. Commun. 2021, 12, 7277. 34907190 10.1038/s41467-021-27565-1 PMC8671406

[34] A. Morteza Najarian , M. Vafaie , B. Chen , F. P. García de Arquer , E. H. Sargent , Nat. Rev. Phys. 2024, 6, 219.

[35] B. Sun , A. M. Najarian , L. K. Sagar , M. Biondi , M. J. Choi , X. Li , L. Levina , S. W. Baek , C. Zheng , S. Lee , Adv. Mater. 2022, 34, 2203039. 10.1002/adma.202203039 35767306

[36] a) C. Wood , V. Harrap , W. Kane , Phys. Rev. 1961, 121, 978;

[37] A. Sahu , L. Qi , M. S. Kang , D. Deng , D. J. Norris , J. Am. Chem. Soc. 2011, 133, 6509. 21486029 10.1021/ja200012e

[38] a) M. T. Jiang , Q. Yang , J. L. Xu , Y. Yuan , J. Y. Zhang , Y. N. Zhong , X. Gao , S. D. Wang , Adv. Opt. Mater. 2023, 11, 2202990;

[39] S. Zhang , C. Bi , T. Qin , Y. Liu , J. Cao , J. Song , Y. Huo , M. Chen , Q. Hao , X. Tang , ACS Photonics 2023, 10, 673.

[40] a) S.‐W. Baek , O. Ouellette , J. W. Jo , J. Choi , K.‐W. Seo , J. Kim , B. Sun , S.‐H. Lee , M.‐J. Choi , D.‐H. Nam , ACS Energy Lett. 2018, 3, 2908;

[41] M. Frisch , G. Trucks , H. Schlegel , G. Scuseria , M. Robb , J. Cheeseman , G. Scalmani , V. Barone , G. Petersson , H. Nakatsuji , Inc., Wallingford CT 2016.

[42] L. Dou , Y. Yang , J. You , Z. Hong , W.‐H. Chang , G. Li , Y. Yang , Nat. Commun. 2014, 5, 5404. 25410021 10.1038/ncomms6404

