# Correction to “Silver Telluride Colloidal Quantum Dot Solid for Fast Extended Shortwave Infrared Photodetector”

**DOI:** 10.1002/advs.202508611

**Published:** 2025-06-09

Ahn, Yongnam, et al. “Silver Telluride Colloidal Quantum Dot Solid for Fast Extended Shortwave Infrared Photodetector.” *Advanced Science* 11.44 (2024): 2407453.

1) Several grammatical errors were found in the Results and Discussion section. The following sentences need to be revised accordingly:

- In the fourth paragraph of page 1, there is a grammatical error. The sentence “*…, resulting in a reduced trap states and increased carrier mobility*” should be revised to “*…, resulting in reduced trap states and increased carrier mobility*.”
- In the caption of Figure 1c, “*XRD pattern of Ag_2_Te CQDs (top)…*” should be revised to “*XRD patterns of Ag_2_Te CQDs (top)…*”
- In the second paragraph of page 3, there is a grammatical error. The sentence “*…suggest that all the ligands are predominantly bound in the Ag–thiolate form.^[15]^*” should be revised to “*…suggests that all the ligands are predominantly bound in the Ag–thiolate form.^[15]^*”
- In the second paragraph of page 7, there are grammatical errors. The sentence “*…demonstrated a f_−3 dB_ of 100 kHz, whereas the MPA‐CQD devices exhibited a f_−3 dB_ exceeding 102 kHz, which is the measurement limit (*Figure S21, Supporting Information)*.…*” should be revised to “*…demonstrated an f_−3 dB_ of 100 kHz, whereas the MPA‐CQD devices exhibited an f_−3 dB_ exceeding 102 kHz, which is the measurement limit (*Figure S21, Supporting Information*).…*”

2) In the first paragraph of page 4, Table S3 (Supporting Information) is mentioned before Table S2 (Supporting Information). To ensure a smoother reading flow, the following two sentences need to be revised accordingly:

- The sentence “*We observed that the area ratio of unbound thiolate to S 2p decreased after ligand exchange (*Table S3, Supporting Information*)*.” should be revised to “*We observed that the area ratio of unbound thiolate to S 2p decreased after ligand exchange (*Table S2, Supporting Information).”
- The sentence “*This result agrees with the FT‐IR spectra results shown in Figure 2A in that the MPA‐CQD solids exhibit a red‐shift that is smaller by 60 cm^−1^ (*Table S2, Supporting Information*)*.” should be revised to “*This result agrees with the FT‐IR spectra results shown in* Figure 2A *in that the MPA‐CQD solids exhibit a red‐shift that is smaller by 60 cm^−1^ (*Table S3, Supporting Information).”

Please note that the order of Tables S2 and S3 (Supporting Information) has also been updated accordingly in the attached PDF file (*SI_Revised_final*). No other changes have been made to the contents of the PDF file.

3) In Figure 4 on page 6, the positions of Figures 4D and 4G have been switched. Therefore, the two panels should be rearranged. Please refer to the attached PowerPoint file (*Main Figure_High resolution_final*) for the correctly arranged version of Figure 4.

After careful review, we have confirmed that the figure caption and the corresponding descriptions in the manuscript are appropriately stated.

4) Several typos were found in the Materials and Characterization sections of the Experimental Section. For consistency in formatting, please revise the following phrases or sentences as indicated below:

- In the Materials section, the phrase “*trioctylphosphine (TOP, tech. grad. 90%)” should be corrected to “trioctylphosphine (TOP, tech. grade. 90%)*.”
- In the Materials section, the sentence “*2‐mercaptoethanol (ME, extra pure) was procured, chloroform, and isopropyl alcohol (IPA, HPLC grade) were procured from Daejung*” should be revised to “*2‐mercaptoethanol (ME, extra pure), chloroform, and isopropyl alcohol (IPA, HPLC grade) were procured from Daejung*.”
- In the Characterization section, the sentence “*a 1550 nm CW laser (MDL‐III−1550‐100 mW diode laser) equipped was utilized with a collimator (FOC‐01‐A)*,” should be corrected to “*a 1550 nm CW laser (MDL‐III−1550‐100 mW diode laser) equipped with a collimator (FOC‐01‐A) was utilized*.”
- In the Characterization section, the sentence “*All device systems are wrapped in aluminum foil before measuring noise current minimize the influence of external electric fields*.” should be revised to “*All device systems are wrapped in aluminum foil before measuring noise current to minimize the influence of external electric fields*.”

5) In the Supporting Information file, minus signs were incorrectly displayed as hyphens. We have corrected them to proper minus signs to improve clarity and ensure consistency with the main text. Please refer to the attached “*SI_Revised_final*” PDF file.

We sincerely apologize for all the errors mentioned.


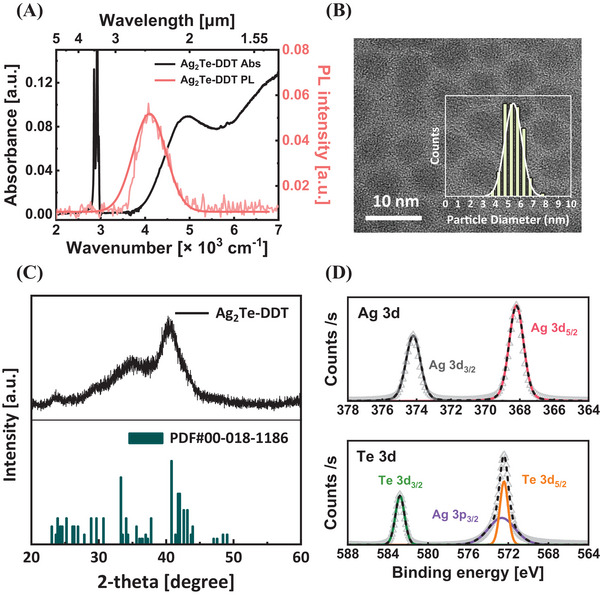


**Figure 1**. Characterization of synthesized Ag_2_Te CQDs. A) Absorbance (black) and infrared PL spectra (pale red) of the DDT‐capped Ag_2_Te CQDs. B) Transmission electron microscopy (TEM) image of DDT‐capped CQDs with a size distribution histogram (inset). C) XRD patterns of Ag_2_Te CQDs (top) and monoclinic Ag_2_Te (bottom) reference. D) XPS spectra of silver (top) and tellurium (bottom) 3d scan of Ag_2_Te CQDs.


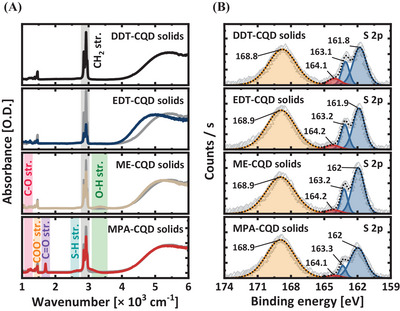


**Figure 2**. Surface passivation of Ag_2_Te CQDs with thiol ligands. A) Absorption spectra of DDT‐ (black), EDT‐ (blue), ME‐ (beige), and MPA‐CQD (red) solids. In each absorption spectrum of ligand‐exchanged CQD solids, the gray solid lines represent the absorption spectrum of DDT‐CQD solids as the baseline. B) XPS spectra of sulfur 2p scan of ligand‐exchanged CQD solids.


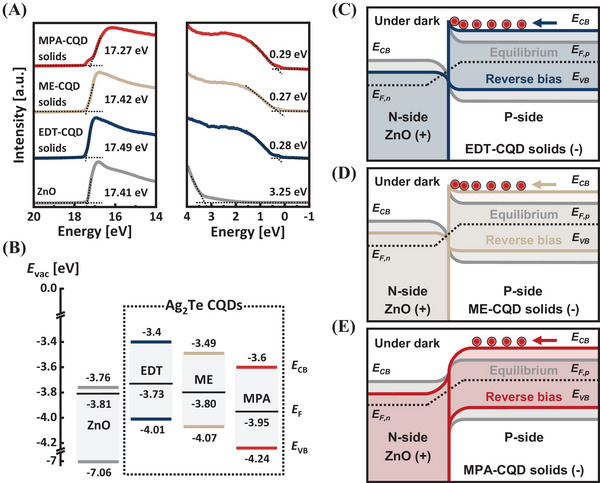


**Figure 3**. Energy band alignment of ligand‐exchanged Ag_2_Te CQD solids. A) Binding energy spectra measured by UPS. B) Energy‐level diagrams for ZnO (gray), EDT‐ (blue), ME‐ (beige), and MPA‐CQD (red) solids from UPS measurements. *E*_vac_, *E*_F_, *E*_CB_, and *E*_VB_ indicate the vacuum level, Fermi level, conduction band edge, and valence band edge, respectively. C–E) Schematic energy level diagrams at the ZnO/CQD solids junction at equilibrium (gray) and under reverse bias (colored) in the dark. *E*_CB_, *E*_VB_, *E*_F,p_, *E*_F,n_ represent the conduction band edge, valence band edge, quasi‐Fermi level of p‐type, and quasi‐Fermi level of n‐type, respectively. Negative charges (red circles) denote thermally generated minority carriers (electrons).


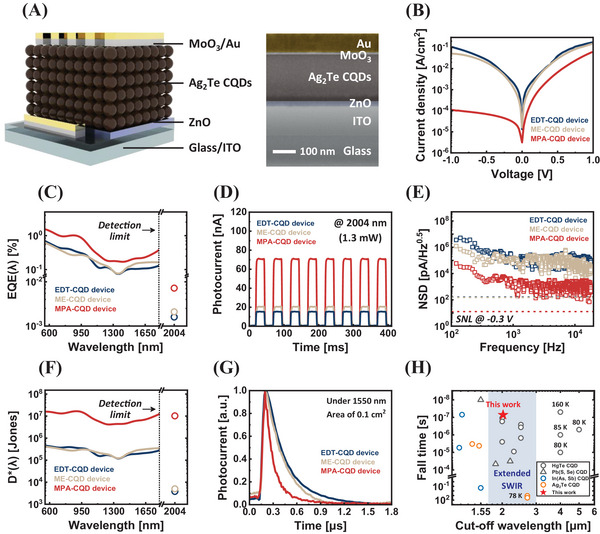


**Figure 4**. Device performance of Ag_2_Te CQD eSWIR photodetectors. A) Device architecture and a cross‐sectional SEM image (scale bar: 100 nm). B) Current density–voltage (*J*–*V*) curves of EDT‐ (blue), ME‐ (beige), and MPA‐CQD (red) devices under dark conditions. C) EQE(λ) spectra of EDT‐ (blue), ME‐ (beige), and MPA‐CQD (red) devices. The dotted line indicates the limit of the instrument (∼1.8 µm). The EQEs below this detection limit were measured directly, while a 2004 nm laser was utilized beyond 1.8 µm. D) On‐off characteristics under short‐circuit conditions with a 2004 nm irradiation of EDT‐ (blue), ME‐ (beige), and MPA‐CQD (red) devices. E) Frequency‐dependent noise spectral density (NSD), and F) *D* ^*^(λ) spectra of EDT‐ (blue), ME‐ (beige), and MPA‐CQD (red) devices. G) Transient photoresponse for the EDT‐ (blue), ME‐ (beige), and MPA‐CQD (red) devices under 1550 nm pulsed irradiation and short‐circuit conditions. H) Figure of merit (FOM) consisting of cut‐off wavelength versus fall time for previously reported CQD‐based photodetectors. The shaded area indicates the eSWIR band (1.7–3.0 µm).

## Supporting information



Supporting Information

