# Supplementary material for: Silver Telluride Colloidal Quantum Dot Solid for Fast Extended Shortwave Infrared Photodetector
Source: Adv Sci (Weinh). 2024 Oct 7;11(44):2407453. doi: 10.1002/advs.202407453 (PMC11600258; doi:10.1002/advs.202407453)
Supplement: Supplementary file 1 — Supporting Information [file ADVS-11-2407453-s001.pdf]

ADVANCED SCIENCE
Open Access

Supporting Information

for *Adv. Sci.*, DOI 10.1002/advs.202407453

Silver Telluride Colloidal Quantum Dot Solid for Fast Extended Shortwave Infrared Photodetector

Yongnam Ahn, So Young Eom, Gahyeon Kim, Jin Hyeok Lee, Beomkwan Kim, Dongeon Kim, Min-Jae Si, Minjung Yang, Yujin Jung, Bo Seon Kim, Yoon Jang Chung, Kwang Seob Jeong and Se-Woong Baek**

Supporting Information

Silver Telluride Colloidal Quantum Dot Solid for Fast Extended Shortwave Infrared Photodetector

Yongnam Ahn†, So Young Eom†, Gahyeon Kim, Jin Hyeok Lee, Beomkwan Kim, Dongeon Kim, Min-Jae Si, Minjung Yang, Yujin Jung, Bo Seon Kim, Yoon Jang Chung, Kwang Seob Jeong, and Se-Woong Baek**

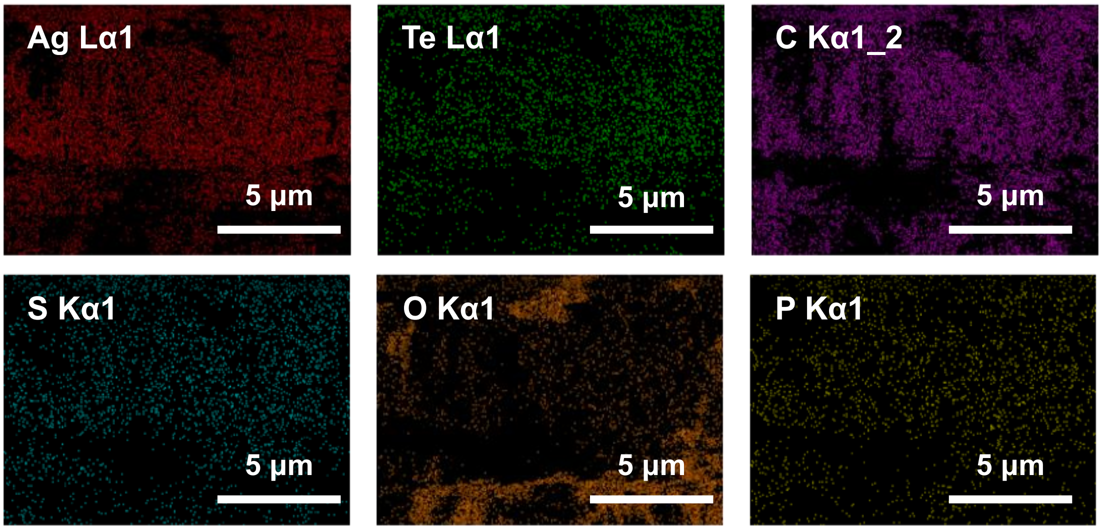

Figure S1. Energy dispersive X-ray spectroscopy (EDS) mapping of DDT-CQD solids.

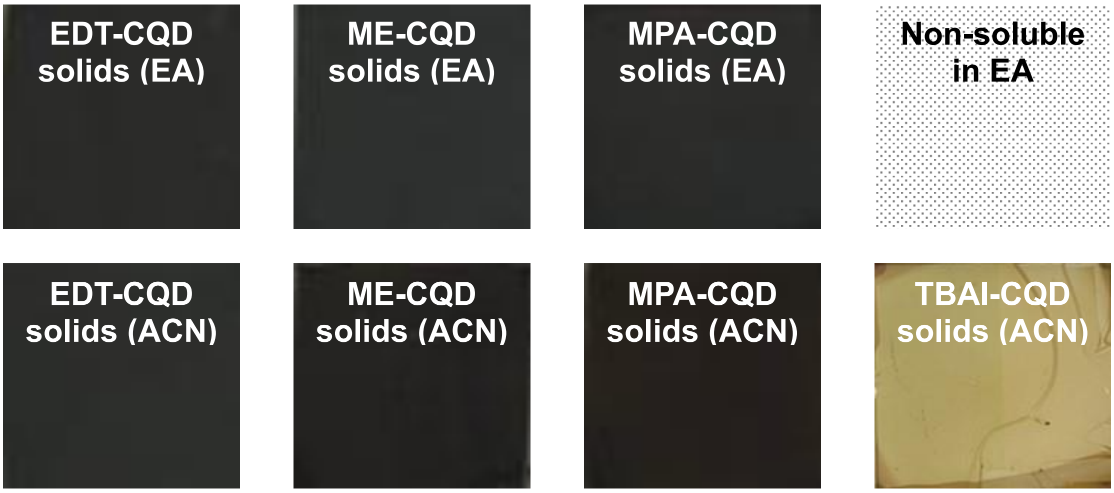

Figure S2. Neat films of CQD solids treated with equivalent molar concentrations and identical procedures using different ligands. Since tetrabutylammonium iodide (TBAI) is not soluble in EA, the ligand exchanges were further performed using acetonitrile (ACN) solvent.

As a result, all ligands successfully achieved ligand exchange in both solvents except for the TBAI ligand, indicating that thiol ligands are more suitable for ligand exchange in this study compared to halide ligands, which have lower binding affinity.

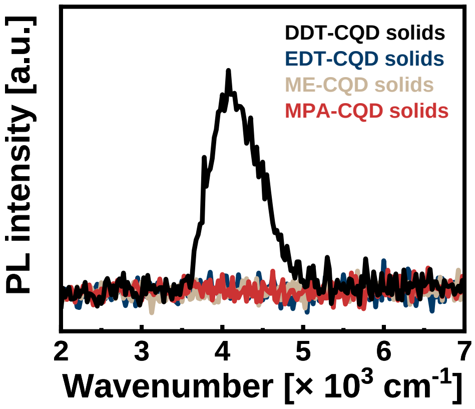

Figure S3. Infrared PL spectra of CQD solids. The black solid line corresponds to DDT-CQD solids, displaying an emission peak at 4100 cm^{-1}. The colored solid lines represent EDT- (blue), ME- (beige), and MPA-CQD (red) solids.

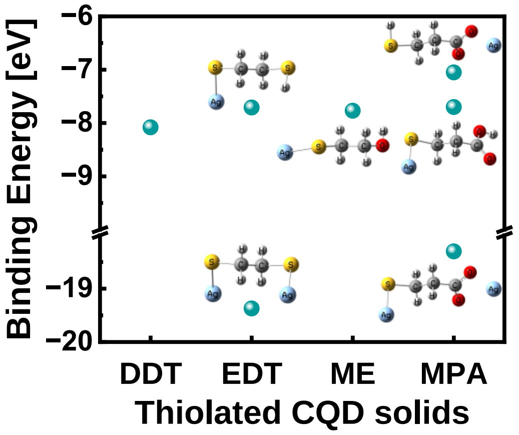

Figure S4. DFT calculations at the B3LYP/LanL2Dz level depicting potential ligand binding to silver atoms in Ag_2Te CQDs. Molecular structures illustrate sulfur (yellow), silver (light blue), carbon (gray), hydrogen (white), and oxygen (red) atoms. Cyan spheres denote binding energy between each ligand and the silver atom.

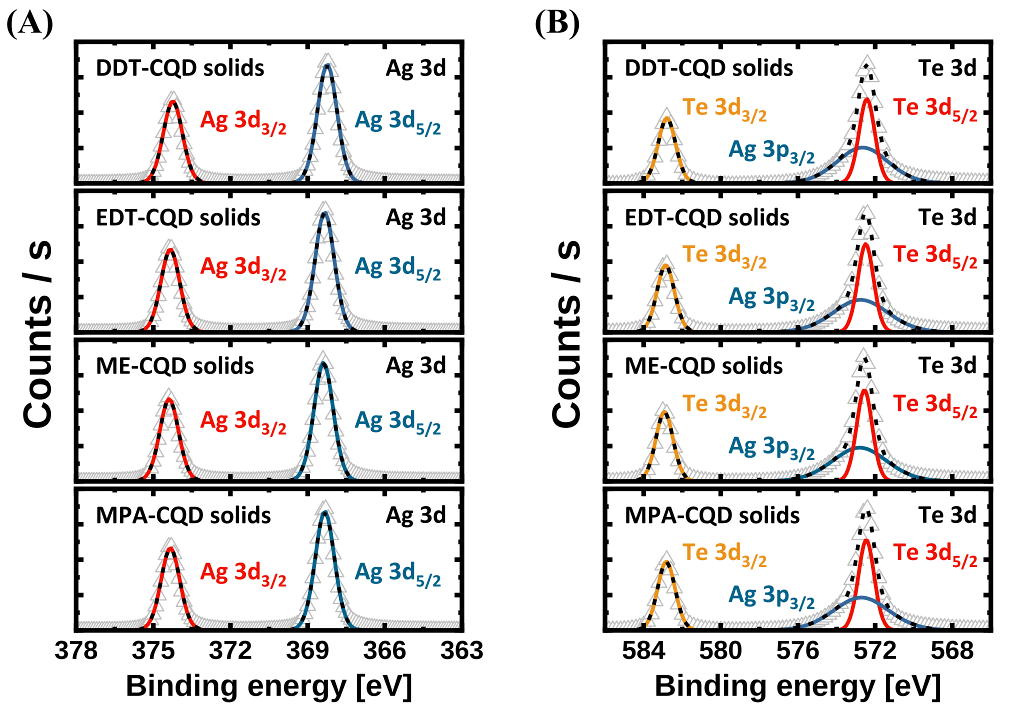

Figure S5. XPS spectra of A) silver and B) tellurium 3d scan of CQD solids before and after ligand exchange.

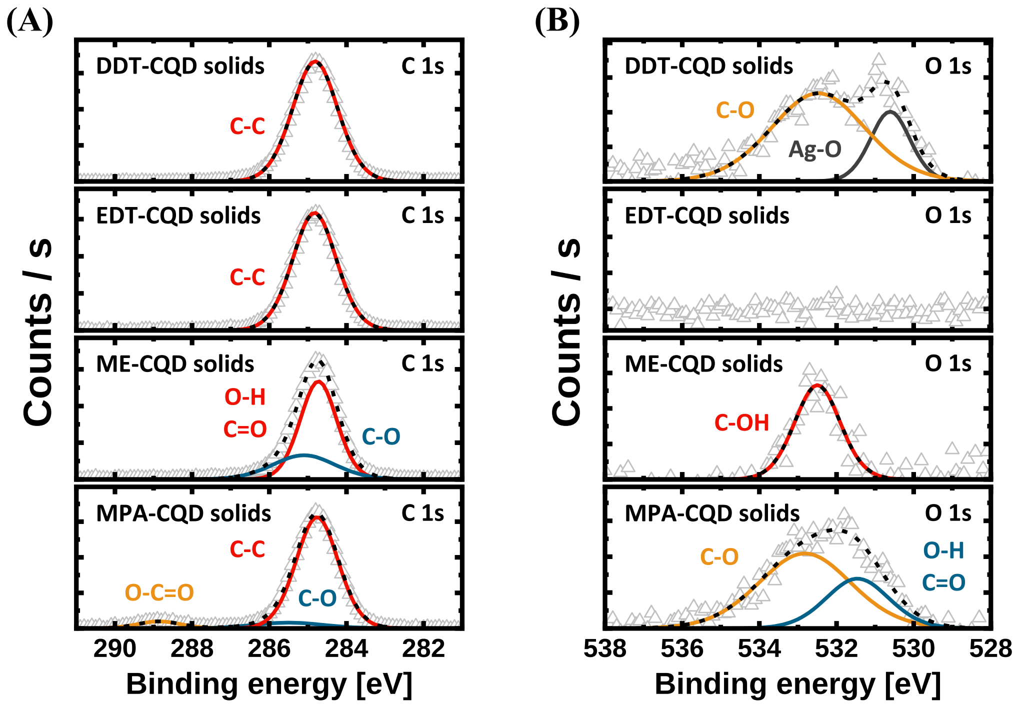

Figure S6. XPS spectra of A) carbon and B) oxygen 1s scan of CQD solids before and after ligand exchange.

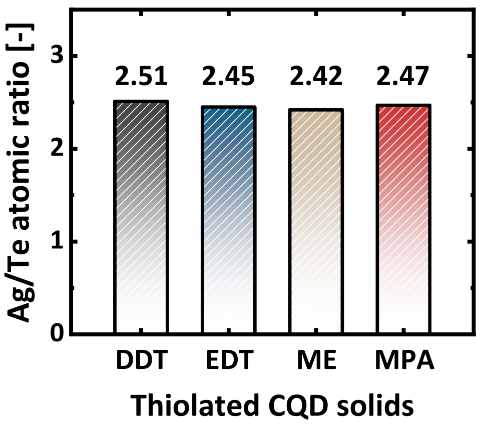

Figure S7. Silver/tellurium atomic ratios of CQD solids before and after ligand exchange, as obtained from XPS analysis.

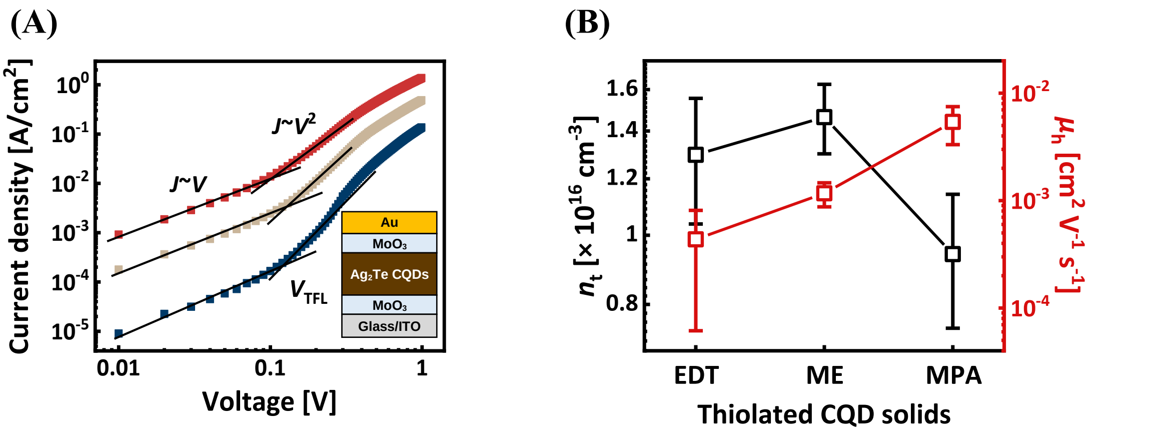

Figure S8. A) *J–V* curves of EDT- (blue), ME- (beige), and MPA-CQD (red) hole only devices (HODs) under dark conditions. V_{TFL} represents the trap-filled-limited voltage. B) Trap densities (n_t, black) and hole mobilities (μ_h, red) of thiolated CQD solids as determined by the SCLC method. Error bars denote standard deviation (N>5).

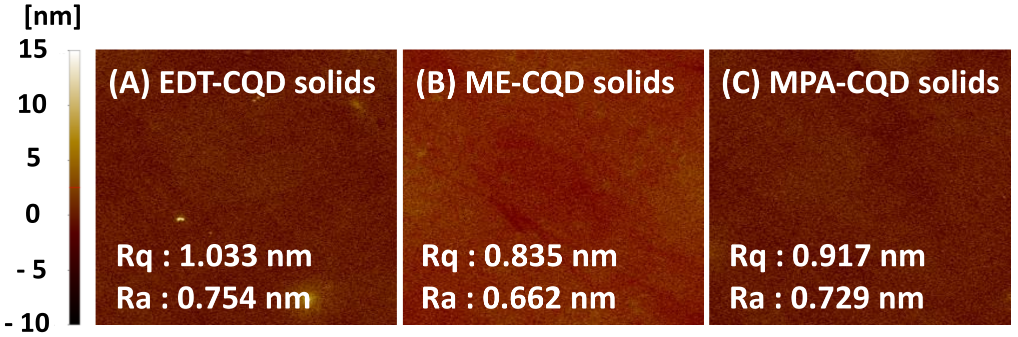

Figure S9. Atomic force microscopy (AFM) images of A) EDT-, B) ME-, and C) MPA-CQD solids. Root mean square (RMS) values are 0.71 nm, 0.51 nm, and 0.56 nm, respectively. The scale bar on the left indicates roughness.

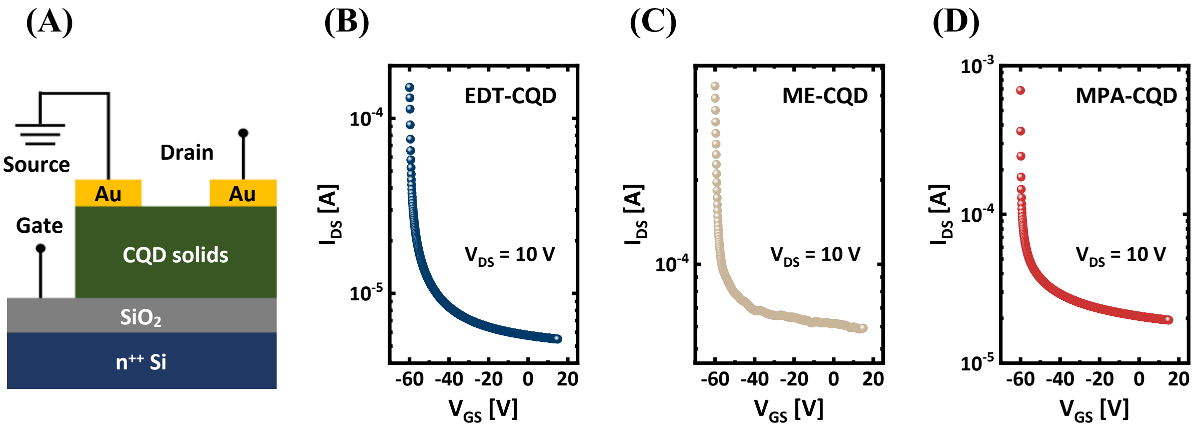

Figure S10. Determination of doping types in ligand-exchanged CQD solids. A) Schematic of FET structure. Transfer curves for B) EDT-, C) ME-, and D) MPA-CQD FET devices. For all FET devices, we noted that the drain current (I_{DS}) increases under constant drain voltage (V_{DS}) when applying a negative gate voltage (V_{GS}). This observation indicates that all CQD solids display p-type characteristics, consistent with our UPS measurements.

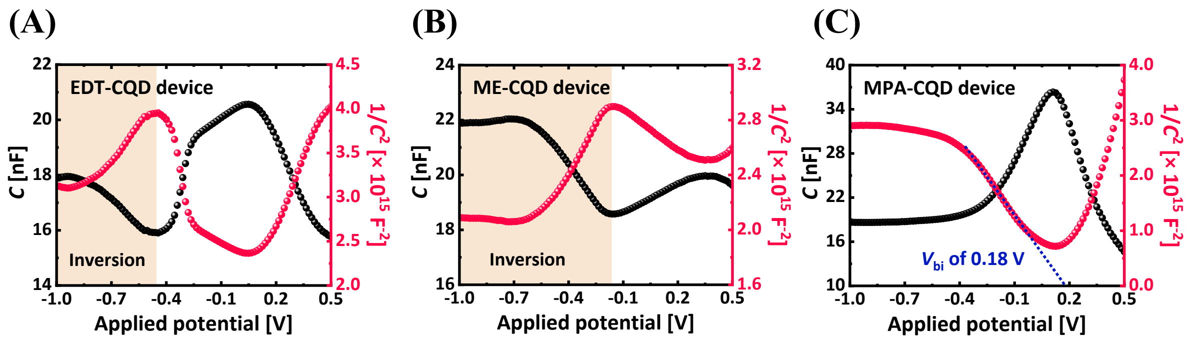

Figure S11. *C*–*V* curves (black circles) and Mott–Schottky ($1/C^2$–V) curves (red circles) for A) EDT-, B) ME-, and C) MPA-CQD devices. V_{bi} stands for built-in potential.

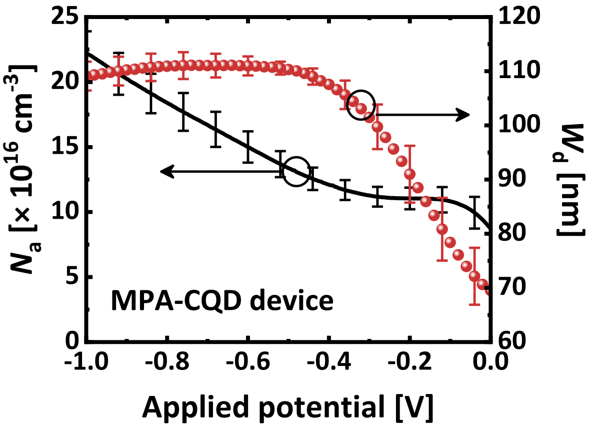

Figure S12. Depletion width (W_d) and acceptor ion concentration (N_a) as a function of applied bias in MPA-CQD devices. Error bars indicate standard deviation (N>5).

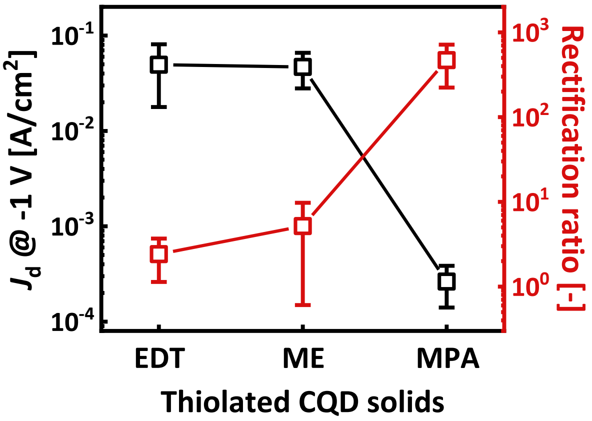

Figure S13. Dark current density (J_d) under dark at -1 V (black) and rectification ratios (red) of EDT-, ME-, and MPA-CQD devices. Error bars represent standard deviation (N>10).

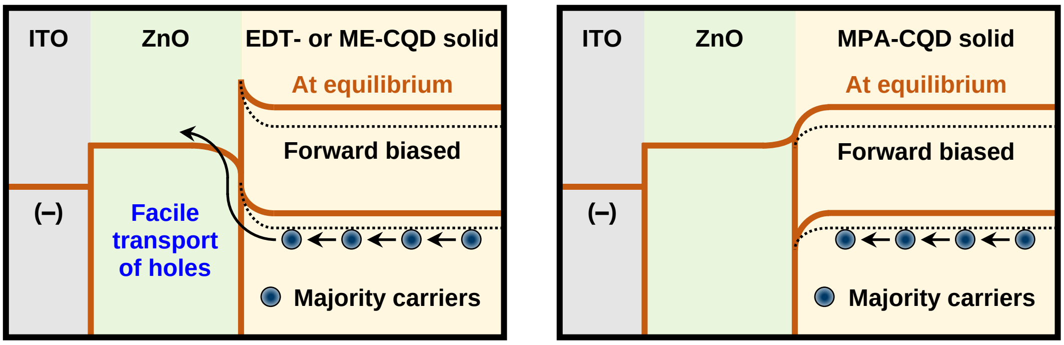

Figure S14. Schematic of energy diagrams under forward bias at the ITO/ZnO/CQD solids junction.

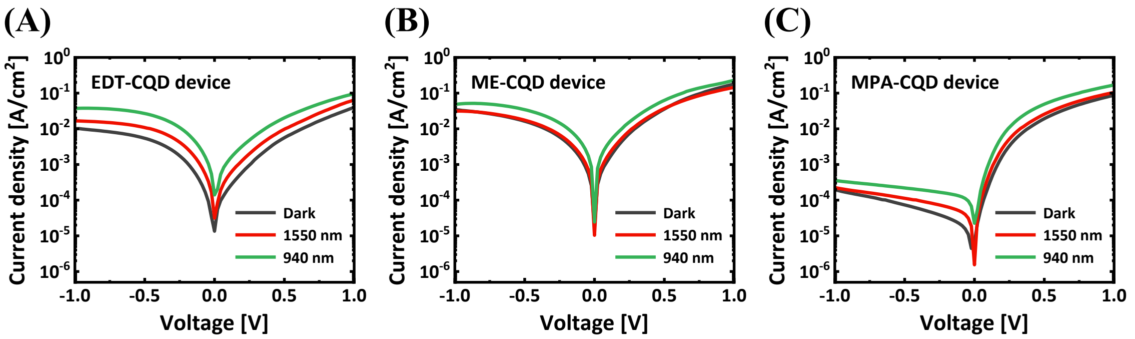

Figure S15. *J–V* curves of A) EDT-, B) ME-, and C) MPA-CQD devices under dark conditions (dark gray), 940 nm laser (green), and 1550 nm laser (red), respectively. The light intensities were 144 mW for 940 nm laser and 94.6 mW for 1550 nm laser, respectively.

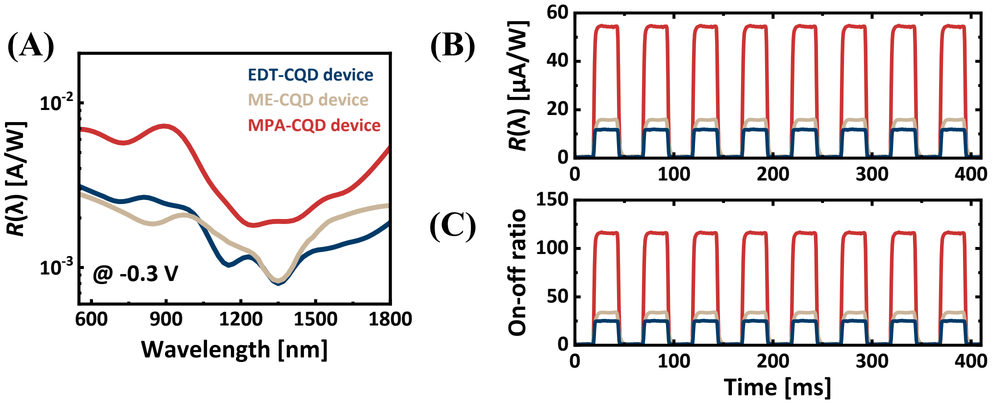

Figure S16. A) R(λ) spectra of EDT- (blue), ME- (beige), and MPA-CQD (red) devices. B) R(λ) and C) On-off ratios of EDT- (blue), ME- (beige), and MPA-CQD (red) devices under 2004 nm irradiation. The on-off ratio is defined as the photocurrent divided by the dark current.

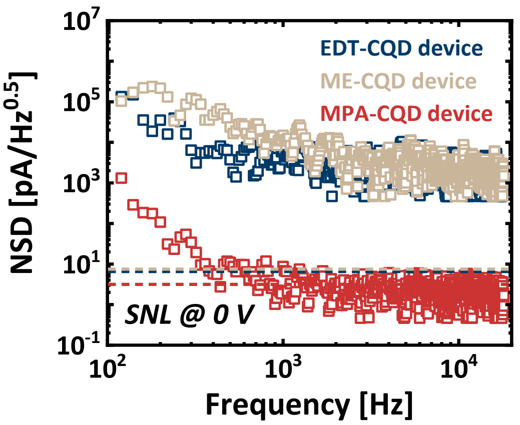

Figure S17. Frequency-dependent noise spectral density (NSD) of EDT- (blue), ME- (beige), and MPA-CQD (red) devices under short-circuit conditions.

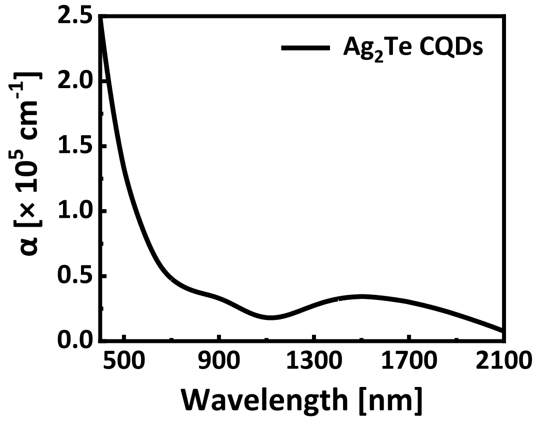

Figure S18. Absorption coefficient (α) of Ag_2Te CQDs. The Ag_2Te CQDs showed α values of 3.4 × 10^4 cm^{-1} at 1550 nm and 1.0 × 10^4 cm^{-1} at 2004 nm. These findings indicate that a micron-thick active layer is required to achieve optimal light absorption at eSWIR wavelengths.

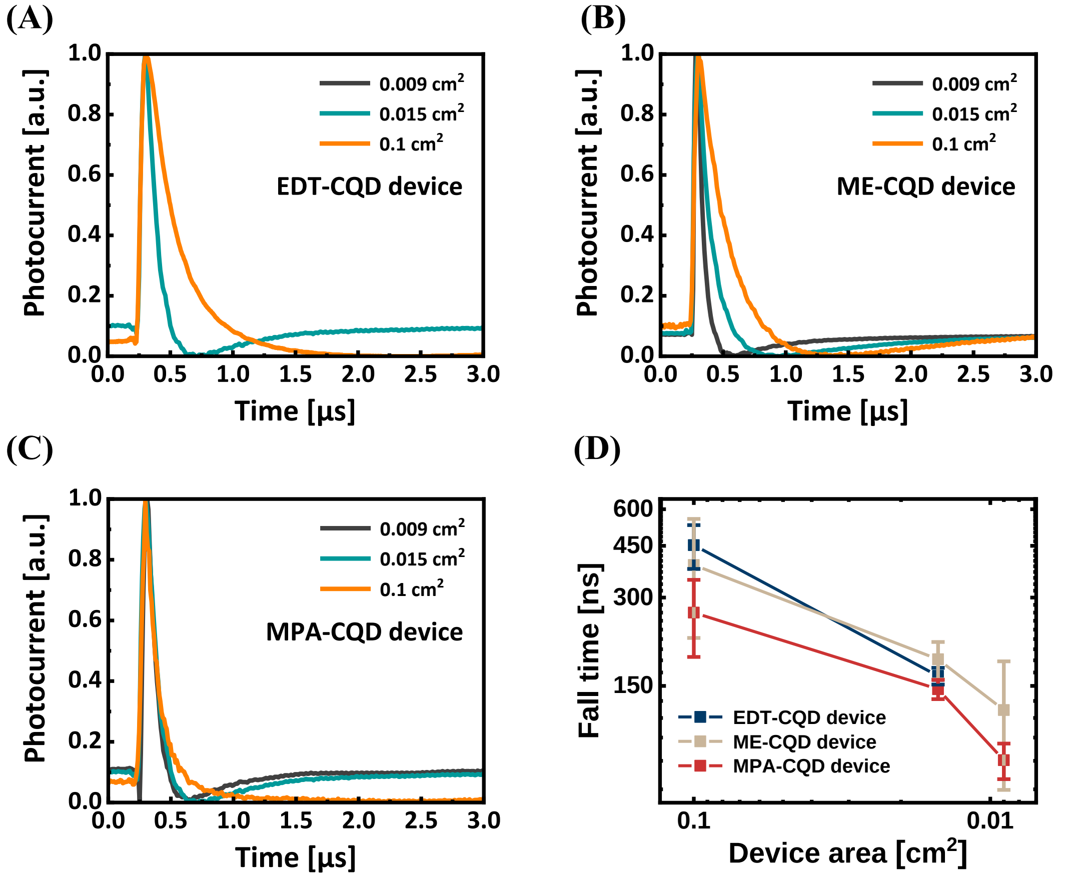

Figure S19. TPC curves of A) EDT-, B) ME-, C) MPA-CQD devices plotted against device area, D) Fall times of EDT- (blue), ME- (beige), and MPA-CQD (red) devices. Error bars

denote the standard deviation (N>5 for an area of 0.009, 0.0015 cm^2, and N>10 for an area of 0.1 cm^2).

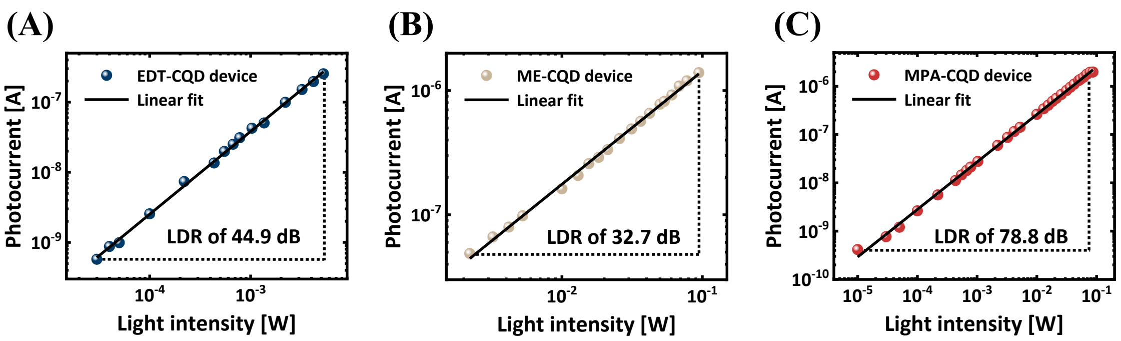

Figure S20. LDR measurements of A) EDT- (blue), B) ME- (beige), and C) MPA-CQD (red) devices under 1550 nm irradiation.

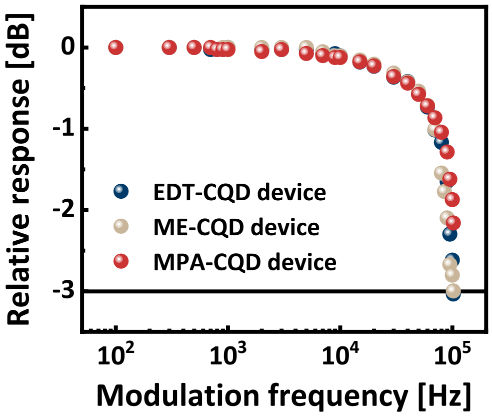

Figure S21. –3 dB bandwidth (f_{-3dB}) measurements of EDT- (blue), ME- (beige), and MPA-CQD (red) devices under 1550 nm irradiation.

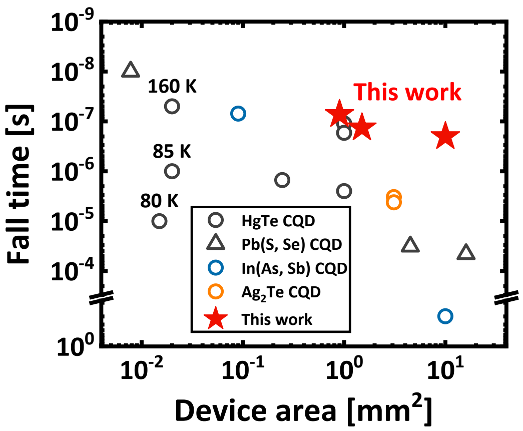

Figure S22. Figure of merit (FOM) consisting of device area versus fall time for previously reported CQD-based photodetectors.

Table S1. Detailed central wavenumber and vibration modes in the FT-IR spectra of ligand-exchanged CQD solids.

Sample	DDT-CQD solids	EDT-CQD solids	ME-CQD solids	MPA-CQD solids
Wavenumber [cm^{-1}] (vibration mode)	1111 (CH_2 wag)	1111 (CH_2 wag)	1043 (C–O stretch)	1190 (C–O stretch)
	1242 (CH_2 rock)	1242 (CH_2 rock)	1377 (C–H bend)	1464 (CH_2 scissor)
	1460 (CH_2 scissor)	1460 (CH_2 scissor)	1466 (CH_2 scissor)	1464 (COO^- stretch)
	2852 (CH_2 sym. stretch)	2852 (CH_2 sym. stretch)	2852 (CH_2 sym. stretch)	1705 (C=O stretch)
	2922 (CH_2 asy. stretch)	2922 (CH_2 asy. stretch)	2922 (CH_2 asy. stretch)	2578 (S–H stretch)
	-	-	3341 (O–H stretch)	2852 (CH_2 sym. stretch)
	-	-	-	2924 (CH_2 asy. stretch)
	-	-	-	3200 (O–H stretch)

Table S2. Absorption peaks of CQD solids before and after ligand exchange.

Sample	Absorption peak (Cut-off wavelength)		
	Before ligand exchange	After ligand exchange	Peak shift
**EDT-CQD solids**	5150 cm^{-1}	4690 cm^{-1}	460 cm^{-1}
**ME-CQD solids**	5220 cm^{-1}	5010 cm^{-1}	210 cm^{-1}
**MPA-CQD solids**	5180 cm^{-1}	5120 cm^{-1}	60 cm^{-1}

Table S3. Binding energies and peak areas in the XPS spectra of sulfur 2p scan.

Sample	Ag–SO_4 or Te 4s	Unbound or free –SH	S $2p_{3/2}$	S $2p_{1/2}$	Area ratio
**DDT-CQD solids**	168.8 eV (20600.7)	164.1 eV (1739.1)	163.1 eV (3770)	161.8 eV (10407.4)	0.12
**EDT-CQD solids**	168.9 eV (29609)	164.2 eV (1580.7)	163.2 eV (5137.9)	161.9 eV (12604.9)	0.089
**ME-CQD solids**	168.9 eV	164.2 eV	163.3 eV	161.9 eV	0.079

	(28278.6)	(1527.6)	(2179)	(14764.7)	
**MPA-CQD solids**	169.0 eV (28488.9)	164.1 eV (965.4)	163.4 eV (2987.4)	162.0 eV (12173.8)	0.066

The area ratio represents the ratio of the 'unbound or free –SH' area to the combined sum of the 'S $2p_{3/2}$ and S $2p_{1/2}$' areas.

Table S4. Parameters obtained from the SCLC method for extraction of hole mobility (μ_h) and trap density (n_t) (N>5).

Sample	V_{TFL} [V]	J_d at V_{TFL} [mA/cm^2]	L [nm]	μ_h [× 10^{-2} cm^2 V^{-1} s^{-1}]	n_t [× 10^{16} cm^{-3}]
**EDT-CQD SCLC device**	0.10 ± 0.02	4.1 ± 4.1	146	0.41 ± 0.4	1.30 ± 0.3
**ME-CQD SCLC device**	0.10 ± 0.01	11.1 ± 3.6	136	1.11 ± 0.4	1.46 ± 0.2
**MPA-CQD SCLC device**	0.12 ± 0.02	28.1 ± 10.5	189	2.81 ± 1.1	0.94 ± 0.2

V_{TFL}, J_d, and L represent the trap-filled-limit voltage, dark current density, and film thickness, respectively. In this table, the term "SCLC device" refers to a hole-only device (HOD).

Table S5. Electric field-related parameters revealed from C–V measurements (N>5).

Sample	V_{bi} [V]	N_a at -0.3 V [cm^{-3}]	W_d at -0.3 V [nm]	L [nm]
**MPA-CQD device**	0.174 ± 0.05	11.28 ± 0.76	101.4 ± 3.72	236

V_{bi} is the built-in potential obtained from the Mott-Schottky curves, N_a is the concentration of the acceptor ion (where the acceptor is Ag_2Te CQDs), W_d is the depletion width, and L is the film thickness. Note that we exclusively calculated parameters for MPA-CQD devices since only MPA-CQD devices exhibit the C–V characteristics of a typical of a p-n junction.

Table S6. Dark current densities (J_d) and rectification ratios of EDT-, ME-, and MPA-CQD devices (N>10).

Sample	J_d at -1 V [mA/cm^2]	J_d at +1 V [mA/cm^2]	Rectification ratio [-]
**EDT-CQD device**	49.4 ± 31.6	123.6 ± 25.2	2.4 ± 1.3 (3.8)
**ME-CQD device**	46.9 ± 19.0	170.9 ± 96.1	5.2 ± 4.6 (15.5)
**MPA-CQD device**	0.26 ± 0.12	85.0 ± 33.1	470 ± 247 (920)

The rectification ratio is defined as J_d at +1.0 V divided by J_d at -1.0 V.

Table S7. Fall time (τ_{fall}) as a function of device area (N>5 for an area of 0.009, 0.015 cm^2, and N>10 for an area of 0.1 cm^2).

Sample	τ_{fall} at 0.1 cm^2 [ns]	τ_{fall} at 0.015 cm^2 [ns]	τ_{fall} at 0.009 cm^2 [ns]
**EDT-CQD device**	453 ± 77 (363)	162 ± 11 (155)	N/A
**ME-CQD device**	387 ± 169 (235)	185 ± 27 (158)	144 ± 65 (99)
**MPA-CQD device**	267 ± 78 (200)	146 ± 11 (134)	84 ± 12 (72)

The τ_{fall} at 0.009 cm^2 values for EDT-CQD devices were not obtained due to the low signal-to-noise ratio.

Table S8. Performance table of SWIR/e-SWIR photodetectors from previous studies.

Photoactive material	Device type	$\lambda_{cut-off}$ [µm]	$D^*(\lambda)$ [Jones]	Fall time [s]	Area [mm^2]	Temp. [K]	Year	Ref.
HgTe CQD	PD	4–5	1.2×10^{11}<br>N/A<br>1×10^{10}<br>3×10^{8}	1×10^{-6}<br>5×10^{-8}<br>N/A	0.02	85<br>160<br>200<br>298	2018	[1]
HgTe NC	PD	5	1.5×10^{9}	5×10^{-7}	N/A	80	2019	[2]
HgTe CQD	PD	2.5<br>5	3×10^{9}<br>7.5×10^{10}	2.6×10^{-7}	1	298	2019	[3]
HgTe CQD	PD (Multi)	1.5–2.5<br>3–5	1×10^{10}<br>1×10^{7}	2.5×10^{-6}	1	150<br>298	2019	[4]
HgTe CQD	PD	2.5	3×10^{9}	3.7×10^{-7}	N/A	298	2019	[5]
PbSe CQD	PD	2.2	1.6×10^{12}	3.2×10^{-5}	4.5	298	2020	[6]
HgTe CQD	PT	2.5	1×10^{11}	1.5×10^{-6}	0.245	298	2020	[7]
PbS CQD	PD	1.55	8×10^{11}	1×10^{-8}	0.0078	298	2021	[8]
HgTe CQD	PD	2	2×10^{10}	1.1×10^{-7}	1	298	2021	[9]
Ag_2Te CQD	PC	2.7	N/A	11.6<br>31	N/A	78<br>298	2021	[10]
PbS CQD	PD	1.85	4.3×10^{13}	4.55×10^{-5}	16	298	2022	[11]
HgTe CQD	PD	4	2×10^{10}	1×10^{-5}	0.015	80	2022	[12]
HgTe CQD	PD	2	2×10^{10}	1.1×10^{-7}	1	300	2022	[13]
HgTe CQD	PD (Multi)	2.1	1.1×10^{11}<br>4.5×10^{11}	N/A	N/A	298	2023	[14]

HgTe CQD	PD	2.3	5×10^{10}	8.92×10^{-6}	N/A	298	2023	[15]
InSb CQD	PD	1.2	1.0×10^{11}	5.4×10^{-6}	N/A	298	2023	[16]
InSb CQD	PD	1.24	4.4×10^{11}	7×10^{-8}	0.09	298	2024	[17]
InSb CQD	PD	1.55	2.7×10^{8}	2.2×10^{-1}	10	298	2024	[18]
Ag_2Te CQD	PD	1.4<br>1.52	3×10^{12}<br>8.8×10^{11}	3.3×10^{-6}<br>4.2×10^{-6}	3.1	298	2024	[19]
Epitaxial HgCdTe	PD	3<br>3.4	$\geq 6 \times 10^{9}$<br>$\geq 5 \times 10^{9}$	$\leq 3.5 \times 10^{-7}$<br>$\leq 2.6 \times 10^{-7}$	0.0025	298	Commercial	[20]
Epitaxial InAs	PD	1.3–3.6	$\geq 3 \times 10^{9}$	3.5×10^{-8}	1	298	Commercial	[20]
Epitaxial InSb	PD	2–5.5	$\geq 1 \times 10^{10}$	≥ 580 kHz	0.28	77	Commercial	[21]
Epitaxial InGaAs	PD	2.1	$\geq 1 \times 10^{11}$	1.8 MHz	0.07	298	Commercial	[21]
**Ag_2Te CQD**	**PD**	**2.0**	**1.0×10^{7}**	**2.0×10^{-7}**<br>**7.2×10^{-8}**	**10**<br>**0.9**	**298**	**This work**	

**CQD: Colloidal quantum dot; PD: Photodiode; PC: Photoconductor; PT: Phototransistor; Multi: Multi-spectral*

Supplementary Note 1. Calculation of trap densities (n_t) and carrier mobilities (μ_h) of Ag_2Te CQD solids using the SCLC method.

In the SCLC method, trap-filled regions are formed by the injection of carriers through forward bias, thereby filling the trap densities present in CQD solids. The trap-filled-limit voltage (V_{TFL}) is defined as the boundary point between the *Ohmic* regime (J~V) and the trap-filled-limit regime. The *SCLC* regime follows Mott–Gurney's law (J~V^2) and was linearly fitted to obtain the trap density (n_t) and hole mobility (μ_h) using the following equations (**Equation S1** and **Equation S2**). [22]

$$n_t = \frac{2V_{TFL}\varepsilon\varepsilon_0}{eL^2} \qquad (1)$$

$$J = \frac{9}{8}\varepsilon\varepsilon_0\mu\frac{(V_{TFL})^2}{L^3} \qquad (2)$$

where, V_{TFL} is the trap-filled-limit voltage, ε_0 is the vacuum permittivity, ε is the relative dielectric constant, e is the elementary charge, L is the thickness of the active layer, J is the current density at V_{TFL}, and μ is carrier mobility. In this method, the L values of each CQD solids were characterized using a surface profiler (Dektak-XT, Bruker). Additionally, we used a dielectric constant of 25 for bulk Ag_2Te. [23]

Supplementary Note 2. Calculation of electric field-related parameters using Mott–Schottky relation.

We determined the V_{bi} for each thiolated device by performing linear fitting on the Mott–Schottky curves in **Figure S11**. The concentration of the acceptor ion (N_a) and the depletion width (W_d) were obtained based on the applied bias using the Mott–Schottky correlation equation shown below (**Equation S3** and **Equation S4**). [8] The values of these parameters at -0.3 V can be found in **Table S5**.

$$\frac{1}{C^2} = \frac{2}{A^2 q\varepsilon\varepsilon_0 N_a}\left(V_{bi} - \frac{k_b T}{q} - V\right) \qquad (3)$$

$$W_d = \frac{1}{N_a}\sqrt{\frac{2\varepsilon\varepsilon_0(V_{bi}-V)}{q(\frac{1}{N_a}+\frac{1}{N_d})}} \qquad (4)$$

where, q is the elementary charge, ε_0 is the vacuum permittivity, and ε is the dielectric constant, which we have taken as 25, representing the bulk Ag_2Te. [23] For the *C*–*V* measurement, the device area (A) of the thiolated devices was 0.1 cm^2. Additionally, k_b represents the Boltzmann constant, T and V denote the absolute temperature and applied bias, respectively. The N_a was determined using **Equation S3**, and the W_d was calculated using **Equation S4**. In this context, N_d represents the concentration of the donor ion, and we assumed 1×10^{18} cm^{-3} for the N_d of ZnO, the electron transport layer. [8]

Supplementary Note 3. Calculation of shot-noise limit (*SNL*) from *J*–*V* curves.

The noise current spectrum is commonly depicted against operating frequency, with the actual noise current level of a photodetector being a combination of frequency-dependent flicker noise, frequency-independent thermal noise, etc. [24] As frequency increases, the noise current level decreases, indicating ideal behavior as it approaches the shot-noise limit (*SNL*) caused by irregular photon arrivals. Therefore, we calculated the *SNL* of each device as follows (**Equation S5**) to compare the actual noise current of devices with the *SNL*, the theoretical minimum noise current.

$$SNL = \sqrt{2qBI_d} \qquad (5)$$

where, q is the elementary charge, B is the noise bandwidth set to 1 Hz, I_d is the dark current, and I_d is obtained from J–V curves under dark conditions.

Supplementary Note 4. Fast response time of Ag_2Te CQD-based photodetectors.

The response time (τ_r) of a photodetector is determined by the RC time constant (τ_{RC}), drift in the space-charge region (τ_{drift}), and diffusion in the charge-neutral region (τ_{diff}) (**Equation S6**) [25] given by:

$$\tau_r = \sqrt{(\tau_{RC})^2 + (\tau_{drift})^2 + (\tau_{diff})^2} \qquad (6)$$

Here, τ_{RC}, proportional to the device area (A) and dielectric constant of the absorbing layer (ε), and inversely proportional to the film thickness (d), is expressed as:

$$\tau_{RC} = 2.2\, RC = \frac{RA\varepsilon\varepsilon_0}{d} \qquad (7)$$

Given that bulk Ag_2Te exhibits a similar ε to other materials such as bulk PbS (~20), [23, 26] similar A and d would result in similar τ_{RC} values to those of other CQD materials. On the other hand, τ_{drift} depends on the junction depletion width (W_d) and carrier mobility (μ), described by:

$$\tau_{drift} = \frac{{W_d}^2}{\mu(V_0 - V_r)} \qquad (8)$$

where, V_0 and V_r are the built-in potential and applied voltage, respectively. We speculate that the Γ-semiconductor property of Ag_2Te, which exhibits a smaller effective mass, enables high conductivity and high μ, as evidenced by the SCLC method. [27] Therefore, we attributed the fast photoresponse of Ag_2Te CQD-based photodetectors to the reduced τ_{drift} resulting from the high μ of Ag_2Te CQD solids.

References

[1] G. H. Kim, S.-H. Kang, J. M. Lee, M. Son, J. Lee, H. Lee, I. Chung, J. Kim, Y.-H. Kim, K. Ahn, *Applied Surface Science* **2023**, 636, 157801.

[2] C. Livache, N. Goubet, B. Martinez, A. Jagtap, J. Qu, S. Ithurria, M. G. Silly, B. Dubertret, E. Lhuillier, *ACS applied materials & interfaces* **2018**, 10, 11880.

[3] X. Tang, M. M. Ackerman, G. Shen, P. Guyot‐Sionnest, *Small* **2019**, 15, 1804920.

[4] X. Tang, M. M. Ackerman, M. Chen, P. Guyot-Sionnest, *Nature photonics* **2019**, 13, 277.

[5] B. Martinez, J. Ramade, C. Livache, N. Goubet, A. Chu, C. Gréboval, J. Qu, W. L. Watkins, L. Becerra, E. Dandeu, *Advanced Optical Materials* **2019**, 7, 1900348.

[6] T. Zhu, Y. Yang, L. Zheng, L. Liu, M. L. Becker, X. Gong, *Advanced Functional Materials* **2020**, 30, 1909487.

[7] Y. Dong, M. Chen, W. K. Yiu, Q. Zhu, G. Zhou, S. V. Kershaw, N. Ke, C. P. Wong, A. L. Rogach, N. Zhao, *Advanced Science* **2020**, 7, 2000068.

[8] M. Vafaie, J. Z. Fan, A. M. Najarian, O. Ouellette, L. K. Sagar, K. Bertens, B. Sun, F. P. G. de Arquer, E. H. Sargent, *Matter* **2021**, 4, 1042.

[9] P. Rastogi, A. Chu, T. H. Dang, Y. Prado, C. Gréboval, J. Qu, C. Dabard, A. Khalili, E. Dandeu, B. Fix, *Advanced Optical Materials* **2021**, 9, 2002066.

[10] G. Kim, D. Choi, S. Y. Eom, H. Song, K. S. Jeong, *Nano Letters* **2021**, 21, 8073.

[11] L. Sheng, C. Yi, L. Zheng, Y. Liu, J. Zheng, X. Gong, *Journal of Materials Chemistry C* **2022**, 10, 2783.

[12] T. H. Dang, C. Abadie, A. Khalili, C. Gréboval, H. Zhang, Y. Prado, X. Z. Xu, D. Gacemi, A. Descamps‐Mandine, S. Ithurria, *Advanced Optical Materials* **2022**, 10, 2200297.

[13] P. Rastogi, E. Izquierdo, C. Gréboval, M. Cavallo, A. Chu, T. H. Dang, A. Khalili, C. Abadie, R. Alchaar, S. Pierini, *The Journal of Physical Chemistry C* **2022**, 126, 13720.

[14] P. Zhao, T. Qin, G. Mu, S. Zhang, Y. Luo, M. Chen, X. Tang, *Journal of Materials Chemistry C* **2023**, 11, 2842.

[15] J. Yang, Y. Lv, Z. He, B. Wang, S. Chen, F. Xiao, H. Hu, M. Yu, H. Liu, X. Lan, *ACS Photonics* **2023**, 10, 2226.

[16] Muhammad, D. Choi, D. H. Parmar, B. Rehl, Y. Zhang, O. Atan, G. Kim, P. Xia, J. M. Pina, M. Li, *Advanced Materials* **2023**, 35, 2306147.

[17] L. Peng, Y. Wang, Y. Ren, Z. Wang, P. Cao, G. Konstantatos, *ACS nano* **2024**, 18, 5113.

[18] S. Jee, M. J. Si, J. H. Choi, D. Kim, C. Kim, D. Yang, S. W. Baek, *Advanced Optical Materials* **2024**, 2303097.

[19] Y. Wang, L. Peng, J. Schreier, Y. Bi, A. Black, A. Malla, S. Goossens, G. Konstantatos, *Nature Photonics* **2024**, 18, 236.

[20] *VIGO Photonics*, http://vigophotonics.com, **2017–2022**.

[21] Hamamatsu Photonics, http://www.hamamatsu.com/eu/en.html, **1948–2023**.

[22] M.-J. Choi, F. P. García de Arquer, A. H. Proppe, A. Seifitokaldani, J. Choi, J. Kim,

S.-W. Baek, M. Liu, B. Sun, M. Biondi, *Nature communications* **2020**, 11, 103.

[23] J. Zhu, R. Pandey, *Journal of Physics and Chemistry of Solids* **2019**, 129, 41.

[24] R. Saran, R. J. Curry, *Nature Photonics* **2016**, 10, 81.

[25] B. Sun, A. M. Najarian, L. K. Sagar, M. Biondi, M. J. Choi, X. Li, L. Levina, S. W. Baek, C. Zheng, S. Lee, *Advanced Materials* **2022**, 34, 2203039.

[26] I. Moreels, G. Allan, B. De Geyter, L. Wirtz, C. Delerue, Z. Hens, *Physical Review B—Condensed Matter and Materials Physics* **2010**, 81, 235319.

[27] a) G. Springholz, in *Molecular Beam Epitaxy*, Elsevier, 2018; b) C. Wood, V. Harrap, W. Kane, *Physical Review* **1961**, 121, 978.
